# Applied potassium negates osmotic stress impacts on plant physiological processes: a meta-analysis

**DOI:** 10.1093/hr/uhae318

**Published:** 2024-11-18

**Authors:** Linxing Zhu, Yuming Sun, Rongfeng Wang, Jixing Zeng, Jia Li, Mengting Huang, Min Wang, Qirong Shen, Shiwei Guo

**Affiliations:** Jiangsu Provincial Key Lab for Solid Organic Waste Utilization, Key Lab of Organic-Based Fertilizers Of China, Jiangsu Collaborative Innovation Center for Solid Organic Wastes, Educational Ministry Engineering Center of Resource-saving fertilizers, Nanjing Agricultural University, Nanjing 210095, China; Jiangsu Key Laboratory for the Research and Utilization of Plant Resources/The Jiangsu Provincial Platform for Conservation and Utilization of Agricultural Germplasm, Institute of Botany, Jiangsu Province and Chinese Academy of Sciences (Nanjing Botanical Garden, Memorial Sun Yat-Sen), Nanjing 210014, China; Jiangsu Provincial Key Lab for Solid Organic Waste Utilization, Key Lab of Organic-Based Fertilizers Of China, Jiangsu Collaborative Innovation Center for Solid Organic Wastes, Educational Ministry Engineering Center of Resource-saving fertilizers, Nanjing Agricultural University, Nanjing 210095, China; Jiangsu Provincial Key Lab for Solid Organic Waste Utilization, Key Lab of Organic-Based Fertilizers Of China, Jiangsu Collaborative Innovation Center for Solid Organic Wastes, Educational Ministry Engineering Center of Resource-saving fertilizers, Nanjing Agricultural University, Nanjing 210095, China; Jiangsu Provincial Key Lab for Solid Organic Waste Utilization, Key Lab of Organic-Based Fertilizers Of China, Jiangsu Collaborative Innovation Center for Solid Organic Wastes, Educational Ministry Engineering Center of Resource-saving fertilizers, Nanjing Agricultural University, Nanjing 210095, China; Jiangsu Provincial Key Lab for Solid Organic Waste Utilization, Key Lab of Organic-Based Fertilizers Of China, Jiangsu Collaborative Innovation Center for Solid Organic Wastes, Educational Ministry Engineering Center of Resource-saving fertilizers, Nanjing Agricultural University, Nanjing 210095, China; Jiangsu Provincial Key Lab for Solid Organic Waste Utilization, Key Lab of Organic-Based Fertilizers Of China, Jiangsu Collaborative Innovation Center for Solid Organic Wastes, Educational Ministry Engineering Center of Resource-saving fertilizers, Nanjing Agricultural University, Nanjing 210095, China; Jiangsu Provincial Key Lab for Solid Organic Waste Utilization, Key Lab of Organic-Based Fertilizers Of China, Jiangsu Collaborative Innovation Center for Solid Organic Wastes, Educational Ministry Engineering Center of Resource-saving fertilizers, Nanjing Agricultural University, Nanjing 210095, China; Jiangsu Provincial Key Lab for Solid Organic Waste Utilization, Key Lab of Organic-Based Fertilizers Of China, Jiangsu Collaborative Innovation Center for Solid Organic Wastes, Educational Ministry Engineering Center of Resource-saving fertilizers, Nanjing Agricultural University, Nanjing 210095, China

## Abstract

Potassium (K) availability in plant cells is critical for maintaining plant productivity across many terrestrial ecosystems. Yet, there is no comprehensive assessment of the mechanisms by which plants respond to potassium application in such conditions, despite the global challenge of escalating osmotic stress. Herein, we conducted a meta-analysis using data from 2381 paired observations to investigate plant responses to potassium application across various morphological, physiological, and biochemical parameters under both osmotic and nonosmotic stress. Globally, our results showed the significant effectiveness of potassium application in promoting plant productivity (e.g. +12%~30% in total dry weight), elevating photosynthesis (+12%~30%), and alleviating osmotic damage (e.g. −19%~26% in malonaldehyde), particularly under osmotic stress. Moreover, we found evidence of interactive effects between osmotic stress and potassium on plant traits, which were more pronounced under drought than salt stress, and more evident in C_3_ than C_4_ plants. Our synthesis verifies a global potassium control over osmotic stress, and further offers valuable insights into its management and utilization in agriculture and restoration efforts.

## Introduction

Aggravating climate changes and ineffective irrigation strategies would trigger insufficient soil water availability or excessive salt, subjecting plants on planet to osmotic stresses [1, 2]. These stresses greatly impede plant productivity, posing a substantial threat to agriculture production [3].

Notably, osmotic stresses profoundly influence plant morphology, physiology, and biochemistry [4]. For instance, *Prunus* leaves often exhibit diminished vitality and become curled and wilted under osmotic stresses [5]. Physiologically, this is associated with the raised stomatal closure and reduced NADPH consumption in the Calvin cycle, limiting CO_2_ influx and photosynthetic capacity [5]. Additionally, excessive reduction of ferredoxin due to osmotic stress, leading to the over-accumulation of reactive oxygen species (ROS) in rice leaves, causing oxidative damage to lipids, proteins, and nucleic acid [6]. These events suggest that osmotic stresses significantly impede global agricultural development, highlighting the urgent need to explore strategies to alleviate their detrimental effects on plant physiology and productivity.

Nutrient homeostasis, emerges as a pivotal physiological process enabling plants to resist osmotic stress, given the consistent evidence of nutrient deficiency and the subsequent decline in plant resilience to such stressors [7]. Among these nutrients, potassium (K) limitation is widespread in most terrestrial ecosystems and often becomes a key limiting factor [8]. Numerous studies suggest that K application enhances plant resilience against osmotic stress, whereas the opposite results were observed under K deficiency [9]. For example, K application have been shown to sustain a favorable K^+^/Na^+^ ratio and facilitate nitrogen uptake by wheat even under severe osmotic stress, and shape the evolution of plant adaptation to osmotic stress [10, 11]. However, potassium’s role in defense against osmotic stress extends beyond nitrogen uptake, affecting photosynthesis and oxidative stress in crops like Arabidopsis and bean by inhibiting NADPH oxidase and reducing ROS production [12, 13]. Nonetheless, some studies suggest K application has no significant impact on crop yields under osmotic stress, and in some cases, even reduced yields [14, 15]. These varied responses may be influenced by ecosystem classifications, soil characteristics, and fertilizer compositions [8]. Hence, further research is required to elucidate the general patterns of K management’s impact on plant tolerance to osmotic stress.

When considering the factors influencing K-induced effects, osmotic stress types emerge as a primary determinant. Drought-induced stress results from water supply–demand imbalance [7], while salt-induced stress is associated with Na^+^ accumulation around the root zone [16]. These distinctions highlight the importance of considering the competitive interaction between K^+^ and Na^+^, along with interactions with other ions, when evaluating K effects under salt stress [17]. Additionally, the photosynthetic type of the plant, whether C_3_ or C_4_, may influence its response to osmotic stress and K application. C_4_ plants, favored under high-photorespiration conditions like osmotic stresses [18], enhance water use efficiency and productivity by reducing photorespiration and increasing leaf carbon sequestration [19]. Nevertheless, these observations only demonstrate the specificity of K effects observed in individual experiments. Meta-analysis, a valuable tool for examining plant responses to environmental stresses, has shown promise in recent years by collecting and analyzing available data. For instance, Cooke et al. [20] demonstrates that silicon amplifies its overall constraining impact on both biotic and abiotic stresses by mitigating oxidative damage. Similarly, Zhao et al. [4] highlighted the role of plant growth-promoting rhizobacteria in reducing oxidative damage and boosting plant hormone synthesis during drought. Yet, such analyses have not been used to uncover general patterns of K effects in osmotic stress. Additionally, these investigations have not considered the interaction between beneficial supplements and abiotic stressors, hindering the identification and evaluation of these additives in the context of abiotic stress. Such a meta-analysis will shed new light on whether K modulates osmotic stress effects, contributing to achieving the goals of improving crop yield under stress conditions.

In this study, we conducted a meta-analysis to gain a comprehensive understanding of plant responses to osmotic stress combined with K application. We aim to explore the interaction between K application and osmotic stress on plant growth and physiology and determine whether these interactions are antagonistic or additive. Our hypotheses are: [21] K application generally improves plant resistance to osmotic stresses, with effects varying by stress type [22]. Photosynthetic pathways (C_3_ & C_4_) significantly influence plant performance under osmotic stresses [10]. Antagonistic interactions between osmotic stress and K application on plant behavior are anticipated, with specific patterns depending on stress type and photosynthetic pathways.

## Results

### Global mean responses to potassium and osmotic stress

Compared to CK treatment, osmotic stress (O and OK) negatively impacted plant morphological parameters, including total dry weight (total DW), leaf dry weight (leaf DW), root dry weight (root DW), and leaf area, regardless of K application (Fig. 2a). The photosynthetic rate to transpiration rate ratio (*P_N_*/*T_r_*) remained unchanged under the O treatment compared to CK, while the *P_N_*/*T_r_* ratio was higher under OK treatment than CK (Fig. 2b). However, photosynthetic parameters such as photosynthetic rate (*P_N_*), transpiration rate (*T_r_*), stomatal conductivity (*g_s_*), and chlorophyll significantly declined under both O and OK (Fig. 2b). As shown in Fig. 2c, osmotic parameters were also significantly influenced by O and OK, with increases in electrolyte leakage, water potential, and osmotic potential, while turgor pressure and leaf relative water content (RWC) decreased (Fig. 2c). Additionally, both O and OK reduced the K^+^/Na^+^ ratio and increased Na^+^ content in leaf and root tissues, but only O reduced K^+^ content, while OK did not. Osmotic stress positively influenced antioxidant enzyme activities and active substance contents under both K application and deficiency, but showed distinct effects on soluble protein content (Fig. 2e and f).

K application significantly enhanced plant total DW compared to the control, regardless of osmotic stress (K versus CK, OK versus O; Fig. 2a). Notably, when assessing under osmotic stress, Poaceae and Solanaceae plants showed a stronger yield response to K than Fabaceae plants (Fig. S1). As shown in Fig. 2b, K application positively affected photosynthetic parameters, including *P_N_*, *g_s_*, *T_r_*, and chlorophyll content, but not *C_i_*, with *P_N_* and *g_s_* responding more strongly under osmotic stress. Further analysis indicated that the effects of K application on osmotic parameters were environment dependent. K application reduced water potential, osmotic potential, and electrolyte leakage under osmotic stress, but increased turgor pressure, osmotic potential, and RWC under nonstress conditions (Fig. 2c). When concerning plant K^+^ and Na^+^ contents, both K^+^ content and K^+^/Na^+^ ratio increased by K application, while a decrease was observed in Na^+^ content (Fig. 2d). Antioxidant enzyme activities, except glutathione peroxidase (GPX), were significantly increased by K application under both stress and nonstress conditions (Fig. 2e). Glutathione (GSH), Pro, phenols, and amino acids increased with K application under both osmotic and nonosmotic stress, while malonaldehyde (MDA) and oxidized glutathione (GSSG) showed the opposite trend. The negative response of H_2_O_2_ to K application was only significant under osmotic stress (Fig. 2f).

**Figure 1:**
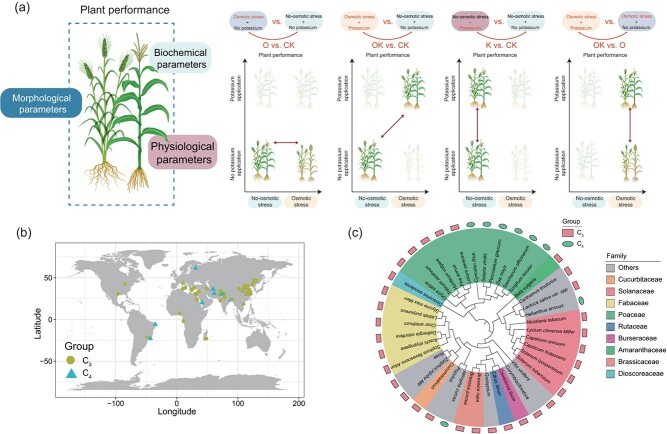
The schematic diagram illustrates the research design, including comparisons between control and treatment conditions (a), geographical distribution of studies in the meta-analysis (b), and plant species analyzed (c).

**Figure 2:**
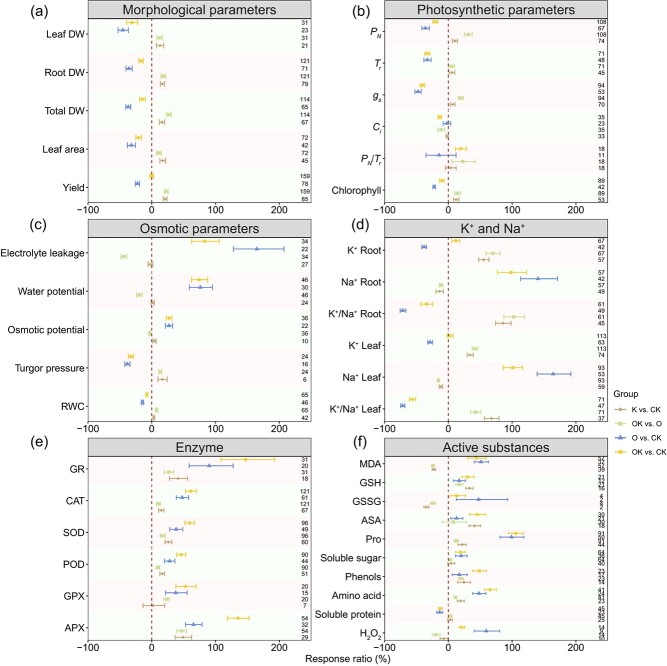
Comparison of the effects of various controls and treatments on plant morphology, physiological parameters, biochemical parameters, including morphological parameters (a), photosynthetic parameters (b), osmosis parameters (c), potassium and sodium ions (d), enzyme activities (e), and active substances (f).The values represent the mean ± 95% confidence intervals. The numerical values on the right side indicate the total count of examined cases. Abbreviations: RWC, leaf relative water content; GR, glutathione reductase; CAT, catalase; SOD, superoxide dismutase; POD, peroxidase; GPX, glutathione peroxidase; APX, ascorbate peroxidase; *P_N_*, photosynthetic rate; *T_r_*, transpiration rate; *g_s_*, stomatal conductivity; *C_i_*, intercellular CO_2_ content; *P_N_*/ *T_r_*, photosynthetic rate/ transpiration rate; MDA, malonaldehyde; GSH, glutathione; GSSG, oxidized glutathione; ASA, ascorbic acid; Pro, proline; H_2_O_2_, hydrogen peroxide; Leaf DW, leaf dry weight; Root DW, root dry weight; Total DW, total dry weight; K^+^ Root, root potassium content; Na^+^ Root, root sodium content; K^+^/Na^+^ Root, the ratio of potassium content to sodium content in root; K^+^ Leaf, leaf potassium content; Na^+^ Leaf, leaf sodium content; K^+^/Na^+^ Leaf, the ratio of potassium content to sodium content in leaf.

**Figure 3:**
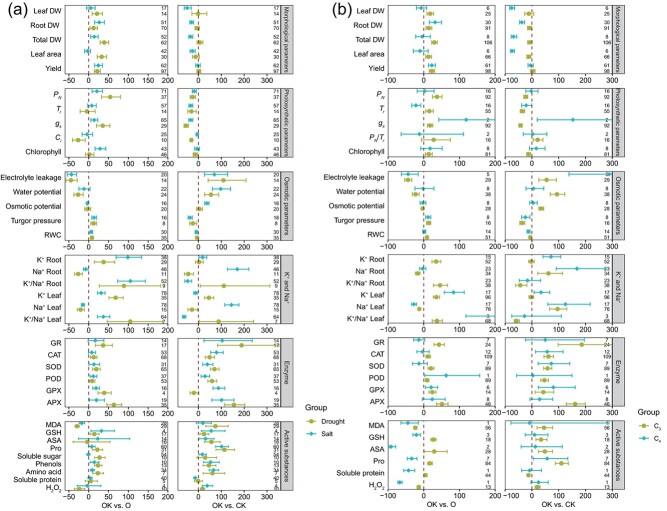
Effects of potassium on physiological and biochemical parameters of plants under osmotic stress, including comparisons of potassium effects under different control and treatment conditions (OK vs. O and OK vs. CK) (a) and between C3 and C4 plants under these conditions (b). Values are means ±95% confidence intervals. The number of observations is shown on the right side. RWC, leaf relative water content; GR, glutathione reductase; CAT, catalase; SOD, superoxide dismutase; POD, peroxidase; GPX, glutathione peroxidase; APX, ascorbate peroxidase; *P_N_*, photosynthetic rate; *T_r_*, transpiration rate; *g_s_*, stomatal conductivity; *C_i_*, intercellular CO_2_ content; *P_N_*/*T_r_*, photosynthetic rate/transpiration rate; MDA, malonaldehyde; GSH, glutathione; GSSG, oxidized glutathione; ASA, ascorbic acid; Pro, proline; H_2_O_2_, hydrogen peroxide; Leaf DW, leaf dry weight; Root DW, root dry weight; Total DW, total dry weight; K^+^ Root, root potassium content; Na^+^ Root, root sodium content; K^+^/Na^+^ Root, the ratio of potassium content to sodium content in root; K^+^ Leaf, leaf potassium content; Na^+^ Leaf, leaf sodium content; K^+^/Na^+^ Leaf, the ratio of potassium content to sodium content in leaf. The numerical values on the right side indicate the total count of examined cases.

### Potassium negates osmotic stress effects based on stress type

Relative to O-drought and CK conditions, OK-drought respectively significantly increased and maintained all plant morphological parameters (Fig. 3a). For photosynthetic parameters, K application raised *P_N_* and *T_r_*, respectively, with reducing *C_i_* under drought (OK-drought vs. O-drought). This pattern contrasts with the OK-drought versus CK group, where all photosynthetic parameters in OK-drought were significantly reduced compared to CK. K application also significantly increased RWC and turgor pressure, while reducing electrolyte leakage and lowering water potential by 26% compared to O-drought, with an opposite trend seen in OK-drought versus CK group. Irrespective of stress conditions, OK-drought increased the K^+^/Na^+^ ratio in both roots and leaves, and elevated leaf K^+^ content while lowering Na^+^ content. Notably, OK-drought significantly reduced MDA and H_2_O_2_ content compared to O-drought, and beyond this, it did not reduce other active substances and enzyme activity relative to O-drought or CK.

In the OK-salt versus O-salt group (Fig. 3a), K application increased total DW, root DW, and yield without reducing leaf DW or leaf area under salt stress. However, OK-salt had significantly stronger negative effects on plant morphological parameters compared to CK. Based on the response ratio, K application under salt stress significantly enhanced *P_N_*, chlorophyll content, and *g_s_*. However, the combined effects of K and salt resulted in a significant decline in these parameters, including *P_N_*, *T_r_*, and *g_s_*, relative to CK conditions. Under salt stress, similar to drought, K application significantly increased RWC and turgor pressure while reducing electrolyte leakage. Notably, compared to CK, OK-salt increased electrolyte leakage and water potential, while turgor pressure and RWC showed the opposite trend. Furthermore, K application under salt stress significantly increased K^+^ content and the K^+^/Na^+^ ratio in leaf and root, while reducing Na^+^ content. In the OK-salt versus CK group, OK-salt treatment significantly elevated root K^+^ and Na^+^ as well as leaf Na^+^, while reducing other parameters. In OK-salt treatment, most enzyme activities and soluble substances either increased (such as catalase (CAT), GPX, and ascorbate peroxidase [APX]) or remained unchanged (such as ascorbic acid [ASA] _OK-salt vs. O-salt_), irrespective of stress conditions. MDA content was significantly reduced in OK-salt compared to O-salt, while soluble protein content was significantly lower compared to CK.

### Potassium negates osmotic stress effects based on plant type

When exposed to osmotic stress, C_3_ plants exhibited more pronounced responsiveness to K application in morphological parameters, particularly root DW, total DW, and leaf DW (Fig. 3b). In contrast, C_4_ plants showed sensitivity to K mainly in root DW and yield (Fig. 3b). C_3_ plants experienced significant improvements in photosynthetic parameters with K, while C_4_ plants displayed variable responses, with unchanged *P_N_* and chlorophyll, a decrease in *T_r_*, and an increase in *g_s_*. Interestingly, K application in C_3_ plants also increased RWC and turgor pressure by 9% and 13%, respectively, while reducing electrolyte leakage and water potential by 44% and 22%, respectively. In C_4_ plants, electrolyte leakage and turgor pressure changes paralleled those in C_3_ plants, with no significant alterations in other parameters. K application significantly reduced root and leaf Na^+^ content in C_3_ plants, with increasing other ion parameters. Similar pattern was observed in C_4_ plants, except for leaf Na^+^. Notably, K had a stronger influence on root K^+^ and leaf K^+^/Na^+^ ratio in C_4_ plants compared to C_3_ plants. In assessing the biochemical distinctions attributable to plant types, the impact induced by K was beneficial in C_3_ plants except for MDA and H_2_O_2_, while only GPX and POD exhibited a positive response to K application in C_4_ plants.

In the OK versus CK group, the combined treatment of osmotic stress and potassium had a more pronounced negative impact on the morphological parameters of C_4_ plants than C_3_ plants (Fig. 3b). OK exposure led to a significant decrease in photosynthetic parameters in C_3_ plants, while C_4_ plants remained mostly unaffected, except for a significant increase in *g_s_*. As shown in Fig. 3b, electrolyte leakage in C_4_ plants increased more than in C_3_ plants under OK, especially OK-salt stress (Fig. S2b). Regardless of plant type, leaf turgor pressure and RWC decreased by 24%–36% and 5%–12%, respectively, after OK application. Meanwhile, in OK-C_3_ plants, water potential, and osmotic potential increased by 93% and 34%. Additionally, OK treatment positively affected Na^+^ content in C_3_ plants but reduced the K^+^/Na^+^ ratio, while in C_4_ plants, both K^+^ and Na^+^ increased without altering the K^+^/Na^+^ ratio. As expected, C_3_ and C_4_ plants exhibited distinct responses to OK treatment regarding enzyme activities and active substances. In C_3_ plants, OK induced Pro, MDA, ASA, GSH, H_2_O_2_ accumulation, and increased all antioxidant enzyme activities, whereas in C_4_ plants, only Pro, CAT, superoxide dismutase (SOD), and GPX were significantly increased.

### Interrelationships among plant traits

**Figure 4:**
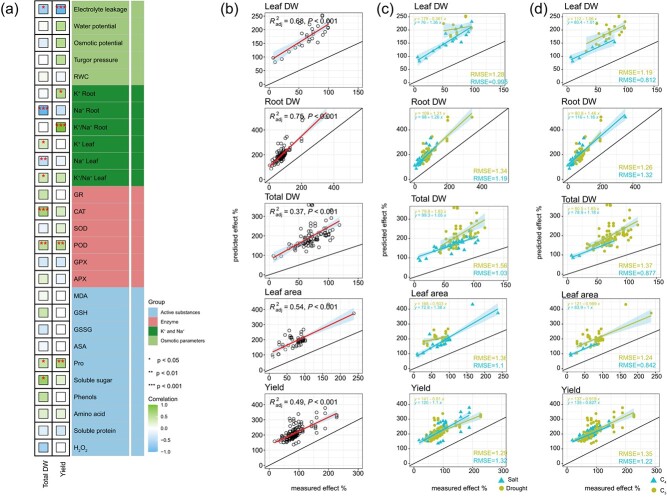
The schematic diagram illustrates (a) correlations between the response ratios of plant total DW and yield with physiological and biochemical parameters under osmotic stresses with K application, and (b-d) relationships between observed (measured effect) and calculated (predicted effect) effects of combined exposure to K and osmotic stress for variables describing plant acclimation responses overall (b), under different stress types (c), and under different plant photosynthetic types (d).All correlation analysis use spearman’s correlation. Correlation coefficient *r* value is shown in the figure, ^*^P < 0.05, ^**^P < 0.01, and ^***^P < 0.001. The black line in b, c, and d indicates a linear fit with a 1:1 correlation. The “RMSE” value in the fitted equation signifies the strength of the interaction. Abbreviations: RWC, leaf relative water content; GR, glutathione reductase; CAT, catalase; SOD, superoxide dismutase; POD, peroxidase; GPX, glutathione peroxidase; APX, ascorbate peroxidase; MDA, malonaldehyde; GSH, glutathione; GSSG, oxidized glutathione; ASA, ascorbic acid; Pro, proline; H_2_O_2_, hydrogen peroxide; Leaf DW, leaf dry weight; Root DW, root dry weight; Total DW, total dry weight; K^+^ Root, root potassium content; Na^+^ Root, root sodium content; K^+^/Na^+^ Root, the ratio of potassium content to sodium content in root; K^+^ Leaf, leaf potassium content; Na^+^ Leaf, leaf sodium content; K^+^/Na^+^ Leaf, the ratio of potassium content to sodium content in leaf.

In our visual analysis, a heatmap was plotted to show the correlations between yield, total DW, and physiological or biochemical characteristics, alongside the computation of correlation coefficients (Fig. 4a). Figure 4a highlights the substantial negative correlations between leaf electrolyte leakage, leaf Na^+^ content and root Na^+^ content and total DW. Conversely, there are significant positive correlations with total DW for soluble sugar, Pro, POD, CAT, leaf K^+^ content, and leaf K^+^/Na^+^ ratio. Furthermore, we observed that the number of plant physiological and biochemical parameters correlating with yield was relatively fewer than those linked to total DW. Among them, only the electrolyte leakage in the leaf exhibited negative correlations with yield. Conversely, significant positive correlations were identified for Pro, POD, the K^+^/Na^+^ ratio in root, and root K^+^ content.

### Plant acclimation responses to exposure to potassium and osmotic stress

In all measured parameters, the combined effects of osmotic stress and K application significantly deviated from the expected trend (Fig. 4b, Fig. S3). Briefly, the cumulative effects of K and osmotic stress did not closely align with the effects of K and osmotic stress when considered individually. Exposure to K application or osmotic stress resulted in decreased or increased levels of stress markers, such as MDA accumulation, respectively (Fig. S3). When plants were exposed to a combination of K application and osmotic stress, the effects were significantly less than additive. This pattern was also observed in the case of total DW, yield, RWC, etc., and is more pronounced.

Moreover, the specific causes of parameter changes align with variations in osmotic stress and photosynthetic plant types. Drought and salt stress exhibited distinct responses to K application, with the RMSE for total DW higher under drought (Fig. 4c). Conversely, the RMSE values for the K^+^/Na^+^ ratio both root and leaf tissues were higher in response to salt stress (Fig. S4). In addition, among the measured morphological parameters, plants exhibited a positive response to K, especially in the C_3_ plants, while osmotic stress had a negative effect (Fig. 2, Fig. S5). The combined impact of K and osmotic stress was nonadditive, with C_3_ plants responding more strongly than C_4_ plants (Fig. 4d). Similarly, the effects on *P_N_* and *T_r_* were less pronounced than expected (Fig. S3, Fig. S6). The osmotic parameters in both C_3_ and C_4_ plants were less influenced by K but critically influenced by osmotic stress. When compared to the cumulative responses expected from the individual effects of K and osmotic stress, the observed reductions in plant osmotic parameters when exposed to a combination of K and osmotic stress were significantly smaller than additive. Notably, C_3_ plants exhibited larger RMSE values for electrolyte leakage. This nonadditive effect was also seen in K^+^ and Na^+^ content, with Na^+^ showing a greater nonadditive effect in C_4_ plants, indicated by a higher RMSE value.

## Discussion

**Figure 5:**
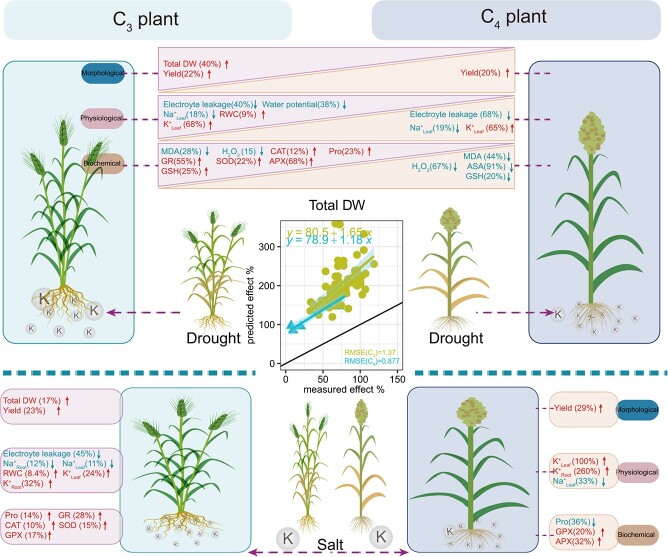
Conceptual figure showing the enrichment (red vertical arrows) and depletion (blue vertical arrows) of functional traits on plant growth in OK relative to O (OK vs. O).

To mitigate growth reduction and yield losses from osmotic stress and K limitation, plants employ K to amplify osmotic stresses signal transduction, activating adaptive mechanisms. Our meta-analysis confirms that K application influences plant resistance to osmotic stress, with effects varying by taxonomic groups due to differences in photosynthetic pathways and stress types. K conferred greater benefits under drought stress compared to salt stress, particularly in C_3_ plants, which showed more significant improvements in morphological traits than C_4_ plants (Fig. 5). Our meta-analysis also revealed notable distinctions in the interaction between K and osmotic stress (drought and salt) in C_3_ and C_4_ plants (Fig. S6). These findings are pivotal for understanding plant responses to osmotic stress with K application, thus enhancing plant tolerance and productivity.

K is a critical nutrient for plant growth and development, with osmotic stress commonly leading to reduced K^+^ content across various species (Fig. 2), including *Catharanthus roseus* [33], wheat [10], and soybean [34]. This deficiency is likely due to significant K^+^ loss from roots, triggered by membrane depolarization and subsequent K^+^ efflux via guard cell outward-rectifying K^+^ channels in the root epidermis [35]. This depolarization is primarily attributed to osmotic stress-induced suppression of the H^+^-ATPase pump and the substantial translocation of sodium ions (Na^+^) across the plasma membrane [26]. While exogenous K^+^ has been shown to restore proton pump activity in some cells [36], its effectiveness under osmotic stress in terrestrial plants remains uncertain. Additionally, K^+^ efflux through ROS-activated channels further contributes to K^+^ loss [37]. Over the past decade, the retention of K^+^ within organisms under osmotic stress has emerged as a novel and critical component of stress tolerance mechanisms. Studies, including Chen et al. [38] on barley, demonstrate a strong correlation between K^+^ retention and osmotic stress tolerance, which has been generalized to other land plants, such as wheat [10] and soybean [34]. These findings support that the nutritional benefits of K can offset osmotic stress-induced reductions in total DW (Fig. 2a), and align with the positive correlation between K^+^ content and total DW (Fig. 4a).

### Potassium helps plants recover more effectively from drought than from salt stress

In contrast to drought, salinity stress induces both osmotic stress and ion toxicity, particularly from Na^+^, which reduces nutrient absorption and damages proteins [17]. A previous report found that maize hybrid 8441 exhibited high Na^+^ accumulation under salt stress, contributing to its sensitivity, while the higher K^+^ content in maize hybrid 26 204 explains its superior salt tolerance [21]. This is due to selective K^+^ uptake and preferential loading of K^+^ over Na^+^ into the xylem [39]. Intriguingly, numerous studies have shown that K application enhances K^+^ uptake and mitigates Na^+^ accumulation in wheat [10], tomato [40], and juvenile mulloway [41] under salt stress, thereby maintaining an elevated K^+^/Na^+^ ratio, which is a hallmark of optimal metabolic processes. This alleviating effect of K^+^ suggests that Na^+^ inhibits growth, at least partially, by disrupting K^+^ metabolism, including transport and compartmentation [17]. Our findings align with this, showing a sharp increase in the K^+^/Na^+^ ratio with K application under salt stress, which may be vital for plant survival (Fig. 3a). Thus, the adaptive accumulation of Na^+^ in plants may reflect a potassium-mediated strategy to cope with salt stress in hostile environments. However, K did not decrease the plant Na^+^ content under the OK-salt stress to the same level observed under CK conditions, even if its competitive interaction with Na^+^ (Fig. 3a). This suggests that with K application plants under salt stress may still retain a strong ability to retain Na^+^, without greatly hindering the transport of Na^+^ from root to leaf. Interestingly, Jiang et al. [42] reported that Na^+^ toxicity in cucumber roots was mitigated by sulfur, which enhanced PM H^+^-ATPase activity and upregulated salt-responsive genes, including *SOS1*, *SOS2*, and *SOS3* [43]. These genes effectively expel excess Na^+^ from the cell. Our data support this role, as sulfur, when applied in combination, alleviates Na^+^ toxicity by reducing Na^+^ influx (Fig. S7).

The lack of K^+^ exacerbates osmotic stress on the photosynthetic apparatus (Fig. 3a), potentially affecting photosystems I and II [44, 45]. The stomata regulate transpiration rates in response to increased physiological water deficit [46]. This did not prevent changes in water potential under salt stress after potassium application (Fig. 3a). While elastic adjustment (increased cell wall elasticity) and osmotic adjustment (lower leaf osmotic potential) are alternative mechanisms for maintaining leaf turgor under water deficit [47, 48]. In the study of eucalyptus, as cell wall elasticity decreased with increasing water deficit, elastic adjustment failed to lower the threshold for leaf turgor loss under stress. In contrast, osmotic adjustment was key to maintaining leaf turgor under low mid conditions [46]. Hence, osmotic potential did not contribute to reducing leaf turgor loss thresholds and may even exacerbate it during OK-salt stress. By contrast, OK-drought showed stable RWC and osmotic potential (Fig. 3a). In addition to robust osmoregulatory capacity, the rigidity of cell walls across the plant system aids in preserving cell volume and maintaining the integrity of the photosynthetic apparatus, potentially promoting leaf gas exchanges under osmotic stress [48, 49]. Unfortunately, this study focused only on permeability parameters, with no data collected on cell wall properties, which remains underexplored. Moreover, it is believed that K application improved leaf photosynthesis in drought-stressed plants by enhancing the storage of osmoregulatory substances, such as soluble sugars, in photosynthetic cells, which help maintain turgor pressure [50]. Strikingly, since plants under drought stress accumulated significantly more osmoregulatory compounds with K application than those under salt stress, indicating that K’s effectiveness may vary depending on the type of osmotic stress (Fig. 3a). Hence, these events suggest that K application under drought, rather than salt stress, may be a prerequisite for achieving total DW similar to those observed in CK plants (Fig. 3a).

Stomatal regulation and carbon sequestration adjust during osmotic stress, supporting ROS accumulation, which damages cellular components [51]. Previous studies have indicated that plant tolerance to salt stress is linked to the activation of antioxidant enzymes, with multiple K^+^-dependent enzymes changing during K resupply [22]. Remarkably, enzymes act as an antidote by converting H_2_O_2_ to H_2_O [44, 52], particularly CAT and GR, indicating OK-drought may offer higher ROS clearance efficiency (Fig. 3a). Consistent with these findings, overexpression of APX, CAT, or dehydroascorbate reductase (DHAR) in transgenic plants such as tobacco [24] and rice [53, 54] has been shown to significantly enhance osmotic stress tolerance. This may increase NADP availability for electron transport, limiting ROS accumulation in leaves under drought stress and reducing H_2_O_2_ and MDA content [52]. These findings support a significant positive correlation between CAT activity and total DW (Fig. 4a).

Plants also use low-molecular-weight antioxidants, such as phenolic compounds and amino acids, to mitigate oxygen free radical damage [55]. Amino acids protect proteins in peanut leaves from oxidation [50], while phenols and flavonoids in cucumber leaves under osmotic stress effectively eliminate ROS, preventing lipid peroxidation and maintaining membrane fluidity [56]. However, K deficiency alters active substance content, as seen in studies where pyruvate kinase activity is reduced in K-deficient plants, lowering pyruvate levels [22]. These findings underscore the crucial role of nonenzymatic antioxidants in protecting metabolically active mesophyll tissues from oxidative stress, especially during OK-drought stress (Fig. 3a).

### C_3_ plants more suitable for potassium application under osmotic stress

In negative response to drought stress, C_3_ plants outperform C_4_ plants, aligning with the consensus that C_4_ plants’ CO_2_ enrichment mechanism boosts adaptability and maintains high carbon fixation, even under osmotic stress [57]. Additionally, C_3_ plant exhibit greater oxidative damage than C_4_ species, as their antioxidant defense system is less effective at preventing osmotic stress-induced cell damage, reflected by increased MDA content (Fig. S5) [58]. However, as stress intensifies or persists, C_4_ plants become increasingly vulnerable to osmotic stress, reducing carbon fixation, and water use efficiency [59]. This diminishes the advantage C_4_ plants hold over C_3_ plants (Fig. S5b) [60]. In agreement with our results, K application reduced Na^+^ less effectively in C_4_ roots under salt stress, thereby specifically enhancing photosynthesis in C_3_ plants (Fig. S2a and b). This may explain the increased total DW observed in C_3_ plants with K application during osmotic stress (Fig. 3b, Fig. S2a). K also enhanced redox balance and osmotic stress resistance in C_3_ plants, activating SOD enzymes and the ASA–GSH cycle, improving ROS scavenging [61]. In contrast, C_4_ plants showed weaker antioxidant responses (Fig. 3b, Fig. S2a), suggesting they rely less on antioxidant defenses for stress mitigation. Interestingly, plant double mutant plants deficient in both cytosolic and thylakoid APX exhibit enhanced salinity tolerance, suggesting that ROS, such as H_2_O_2_, may play a role in activating osmotic stress signals that contribute to improved stress tolerance[62].

### Less-than-additive increases in plant defense and stress occur under combined potassium and osmotic stress treatments

Considering cumulative interactions, our meta-analysis evaluates whether they deviate more negatively or positively from expectations. However, relying solely on such assessments may mislead conclusions regarding impact direction or environmental roles. Thus, we systematically assess cumulative interaction responses, taking both magnitude and direction into account for osmotic stress and K application [31, 63]. The combination of these factors led to a lower effect on plant osmotic parameters (*P_N_*, H₂O₂, RWC) compared to the additive effect (Fig. S3). Consequently, the primary effects of stress exposure, such as elevated H_2_O_2_ production and decreased RWC, may undergo a transformation into secondary stress symptoms, including membrane damage (higher MDA) and impairment of the photosynthetic apparatus (lower *P_N_*) (Fig. 2b and d). Consistently, the effects of osmotic stress and K application on plant biomass did not exhibit an additive effect (Fig. 4b). The role of H_2_O_2_ as a signaling molecule may explain these findings, enhancing defense responses and decoupling stress and defense [63, 64]. Indeed, increases in antioxidants, proline, and morphological parameters were less-than-additive, suggesting that plant defense responses do not follow an additive pattern, leading to a corresponding absence of additive damage.

Current research has yet to fully clarify the regulatory pathways behind plant stress responses and acclimation. We hypothesize that the observed less-than-additive effects stem from induced cross-tolerance mechanisms [63]. When exposure to two environmental factors (potassium as a factor), co-expression of responses may be amplified and/or defense mechanisms may be mobilized more efficiently. In this pattern, there is a reduction in stress levels and less need for further induction of defense responses compared to the individual effects of the two environmental factors. This cross-resistance may explain the similarities between osmotic stress and potassium-induced protective responses, likely mediated by overlapping signaling cascades involving shared transcription factors, hormones, antioxidants, and ROS like POD and SOD [25, 65]. Thus, in this cross-resistance scenario, osmotic stress may improve potassium use efficiency, while K application can induce resistance to osmotic stress. As demonstrated by our findings (Fig. 2a) and Zhang et al. [66], the K effect on total DW was more pronounced under osmotic stress compared to nonosmotic stress. The antagonistic interaction, measured by the RMSE value, was greater between K and drought stress than between K and salt stress, with C_3_ plants showing higher RMSE values than C_4_ plants (Fig. 4b, Fig. S6). This pattern can be attributed to the distinctive physiological characteristics of C_3_ plants (without specialized photosynthetic adaptations) and the relatively simpler nature of drought stress (without Na^+^ toxicity). Therefore, K application can potentially be utilized as part of a more sustainable C_3_ planting system, reducing reliance on supplemental watering.

## Conclusion

In summary, our meta-analysis confirms the positive effects of potassium on plant traits, including photosynthesis, water status and antioxidant enzymes, and thus multiple physiological functions. More importantly, we found that potassium application could attenuate the negative impact of osmotic stress on multiple physiological and biochemical functions of plants, thereby increasing their overall resistance to osmotic stress. While the responses of plant productivity attributes to potassium under drought stress corroborate those under salt stress, they were higher in magnitude than those under salt stress. Moreover, we further revealed that the increase of plant productivity with potassium application under osmotic stress was more pronounced C_3_ plants than in C_4_ plants. Collectively, our findings provided a realistic view of the pivotal role of potassium in mitigating the impacts of osmotic stress, which is vital for promoting plant growth under the dual challenges of a changing climate and potassium limitation. This establishes a foundation for considering potassium as a prospective abiotic agent for remediating osmotic stress in agriculture.

### Limitations

This study confirms that potassium application significantly enhances plant photosynthesis, antioxidant capacity, and biomass accumulation. Regrettably, the gene data obtained from the collected studies were limited. Given the key role of gene interactions in osmotic stress defense, future research should prioritize understanding plant gene expression in response to potassium. Notably, most experiments were conducted under controlled conditions, emphasizing the need to investigate the impact of climatic factors on potassium efficiency, particularly through long-term trials. While our analysis showed significant differences in the potassium responses of C_3_ and C_4_ plants under osmotic stress, the underlying molecular mechanisms remain unclear due to current data limitations. Additionally, further analysis is needed to understand how different plant functional species respond to potassium. Our analysis indicates that different types of potassium have a considerable impact on plant growth; however, we did not find a clear corresponding relationship between plant species and potassium types. Besides, other elements also support plant resistance to osmotic stress, warranting future studies on the interactions between potassium and these elements.

## Materials and methods

From January 1990 to January 2024, peer-reviewed journal articles were systematically searched via the Web of Science (http://www.isiknowledge.com/) to explore the impact of potassium application on plant response to osmotic stresses. The following terms were employed during the search: (water OR salt OR drought OR salinity) AND (plant). Here, drought and salinity were primarily considered as the main sources of osmotic stresses in plants. Due to significant differences between Chinese and English databases, distinct sets of keywords were selected [23]. Additionally, Chinese publications from January 2000 to January 2024 were supplemented by searches using the China National Knowledge Infrastructure (https://www.cnki.net/) with the terms: (drought OR salt OR water stress) AND (plant).

Studies included in our dataset for meta-analysis must meet the following criteria: [21] the plants were subjected to both osmotic stress and control treatments, along with K application and control (K-deficiency) treatments [22]; if a study spans multiple years, we only include measurements from the final year [10]; if a study spans multiple growing seasons, we only include measurements from the most recent growing season [24]; if an article includes multiple independent experiments, like two experiments in different locations, each is treated as a separate study and included in the dataset [25]; studies must report means and sample sizes (*n*), with at least three replicates per experiment [26]; if multiple publications report the same experiment, the most recent study is used. Then, if only standard errors (SE) were provided for plant variables, we calculate standard deviations (SD) using the formula:


\begin{equation*} \mathrm{SD}=\mathrm{SE}\ast \sqrt{n} \end{equation*}

If neither SD nor SE was reported, we estimate SD using the coefficient of variation from the complete dataset, following Bracken’s [27] methodology.

Ultimately, we retrieved 2381 paired observations from 77 published studies (Supporting Information: dataset S1, including 497 on morphological parameters, 1082 on physiology parameters (415 for photosynthetic parameters, 205 for osmotic parameters, and 462 for K^+^ and Na^+^ ion), and 802 on biochemistry parameters (412 for antioxidant enzyme activity and 390 for active substance)). Notably, four groups of response ratios were assessed according to the experimental design (Fig. 1a), namely O versus CK, OK versus CK, K versus CK, and OK versus O, which represent O (with osmotic stress and without K) versus CK (without osmotic stress and K), OK (with osmotic stress and with K) versus CK (without osmotic stress and K), K (without osmotic stress and with K) versus CK (without osmotic stress and K), and OK (with osmotic stress and with K) versus O (with osmotic stress and without K), respectively. Additionally, most studies in this meta-analysis were conducted in the Northern Hemisphere, particularly in Asia, with limited representation from the Southern Hemisphere (Fig. 1b). Based on previous studies, plants were classified into C_3_ and C_4_ photosynthetic types (Fig. 1c).

### Data analysis

To assess the effects of K application, osmotic stress, and their combination on plant traits, the natural log-transformed response ratio (ln RR_ij_) was used as an index, defined as the “effect size” [28], to weigh the effects, calculated using the following formula:


(1) \begin{equation*} \ln\ \mathrm{R}{\mathrm{R}}_{\mathrm{ij}}=\ln \left(\frac{x_t}{x_c}\right)=\ln \left({x}_t\right)-\ln \left({x}_c\right) \end{equation*}

where *x_c_* and *x_t_* represent the mean values of plant traits in the control and treatment groups, respectively.

The variance (*v_ij_*) of each individual observation was calculated as:


(2) \begin{equation*} {\displaystyle \begin{array}{c}{v}_{ij}=\frac{s_t^2}{n_t{x}_t^2}+\frac{s_c^2}{n_c{x}_c^2}\end{array}} \end{equation*}

where *n_t_* and *n_c_* represent the number of replicates while the *s_t_* and *s_c_* represent the SD of treatment (*t*) and control (*c*) groups, respectively.

The weighting factor (*w_ij_*) for each observation was calculated as the inverse of its variance (*v_ij_*)*:*


(3) \begin{equation*} {\displaystyle \begin{array}{c}{w}_{ij}=\frac{1}{v_{ij}}\end{array}} \end{equation*}

To calculate the overall effects of the treatment group compared to the control group, the “mean effect size” (ln *RR*_++_) was determined as follows:


(4) \begin{equation*} {\displaystyle \begin{array}{c}\ln{RR}_{++}={{\sum}_{i=1}^m{\sum}_{j=1}^{n_j}{w}_{ij}\ln\ R{R}_{ij}}\!\left/ {{\sum}_{i=1}^m{\sum}_{j=1}^{n_j}{w}_{ij}}\right.\end{array}} \end{equation*}

*n* denoted the number of observations in each corresponding comparison group, while m indicated the number of groups.

Percentage values derived from the mean effect size were used to express the impact of K application on plants subjected to both osmotic and nonosmotic stress:


(5) \begin{equation*} {\displaystyle \begin{array}{c}\ln{RR}_{++}\left(\mathrm{expressed}\ \mathrm{in}\ \mathrm{percentage}\right)=\left({e}^{\ln{RR}_{++}}-1\right)\ast 100\%\end{array}} \end{equation*}

A random-effects model was used to estimate mean effect sizes, with 95% confidence intervals (CI) generated through 999 bootstrapped iterations, all conducted via the “metafor” package in R 3.2.1. The determination of the significance of treatment relative to the control is based on whether the 95% confidence interval for the effect size of a parameter crosses the zero point of the horizontal coordinate.

### Mapping of plant phylogenetic trees

After determining the classification of each plant, we constructed a phylogenetic tree using the “V. Lomaker 2” package in the R programming language [29]. Subsequently, we utilized the iTOL (https://itol.embl.de/) to enhance its visual presentation.

### Calculate antagonism, synergy, and additivity

The impacts of osmotic stress and K application on plants were quantified as percentage changes from an untreated baseline. The measured effects of the combined treatment of osmotic stress and K application were compared with the sum of these relative changes [30, 31]. Hence, the predicted values were calculated as the sum of the effects of the two treatment factors (osmotic stress and K application). Additivity is inferred when measured outcomes match the predicted sum of individual treatments; synergy is identified when outcomes surpass this sum. Conversely, outcomes falling below this sum indicate antagonistic interactions. To assess the interaction between K and osmotic stress, observed values (*y*-axis) were plotted against predicted values (*x*-axis) for individual variables using linear regression, following Piñeiro et al. [32]. Differences between the slope (a1) and intercept (b1) of the best-fitting linear regression and the ideal 1:1 line (where a0 = 1, b0 = 1) were evaluated using *t*-tests. We also calculated the root mean square error (RMSE) for both the 1:1 line and the best linear fit to assess model accuracy.

## Code availability

All scripts used in this study can be obtained by contacting the first author.

## Supplementary Material

Web_Material_uhae318

## Data Availability

All data supporting the findings of this article are available within the article and its online supplementary material.
