# Supplementary material for: Applied potassium negates osmotic stress impacts on plant physiological processes: a meta-analysis
Source: Hortic Res. 2024 Nov 18;12(2):uhae318. doi: 10.1093/hr/uhae318 (PMC11825146; doi:10.1093/hr/uhae318)
Supplement: Web_Material_uhae318 [file web_material_uhae318.zip › Supporting File.docx]

**Applied potassium negates osmotic stress impacts on plant physiological processes: a meta-analysis**

Linxing Zhu^a^, Yuming Sun^b^, Rongfeng Wang^a^, Jixing Zeng^a^, Jia Li^a^, Mengting Huang^a^, Min Wang^a^*, Qirong Shen^a^, Shiwei Guo^a^

^a^Jiangsu Provincial Key Lab for Solid Organic Waste Utilization, Key Lab of Organic-Based Fertilizers Of China, Jiangsu Collaborative Innovation Center for Solid Organic Wastes, Educational Ministry Engineering Center of Resource-saving fertilizers, Nanjing Agricultural University, Nanjing 210095, China

^b^Jiangsu Key Laboratory for the Research and Utilization of Plant Resources / The Jiangsu Provincial Platform for Conservation and Utilization of Agricultural Germplasm, Institute of Botany, Jiangsu Province and Chinese Academy of Sciences (Nanjing Botanical Garden, Memorial Sun Yat-Sen), Nanjing, China

*Corresponding author: Min Wang

*E-mail address*: minwang@njau.edu.cn

Tel.: +86-25-8439-5212

**Materials and methods**

The data were extracted from the tables directly or from the figures using Web Plot Digitizer version 4.4. The following plant parameters data, including morphological parameters, physiological parameters, and biochemical parameters, were extracted from each paired observation. Finally, our dataset includes 77 published studies, with 62 in English and 15 in Chinese, comprising 2381 paired observations. Amongst, the observed values of various plant morphological parameters, including leaf dry weight, root dry weight, total dry weight, leaf area, and yield. Additionally, plant physiological parameters, encompassing root Na^+^, root K^+^, the root K^+^ to Na^+^ ratio (K^+^/Na^+^), leaf K^+^, leaf Na^+^, the leaf K^+^ to Na^+^ ratio, photosynthetic rate (*P_N_*), transpiration rate (*T_r_*), stomatal conductance (*g_s_*), intercellular CO_2_ content (*C_i_*), the photosynthetic rate to transpiration rate ratio (*P_N_*/ *T_r_*), chlorophyll, electrolyte leakage, water potential, osmotic potential, turgor pressure, and leaf relative water content (RWC), were also recorded. Critical plant biochemical parameters, including malondialdehyde (MDA), glutathione (GSH), glutathione oxidized (GSSG), ascorbic acid (ASA), proline (Pro), soluble sugar, phenols, amino acid, soluble protein, hydrogen peroxide (H_2_O_2_), gluathione reductase (GR), catalase (CAT), superoxide dismutase (SOD), peroxidase (POD), glutathione peroxidase (GPX), and ascorbate peroxidase (APX), are encompassed by our dataset. For each study, we extracted supporting information, including the latitude and longitude of each experimental site, the K fertilizer types, the plant taxonomic classification, as well as the plant photosynthetic pathway types (C_3_ or C_4_) (supporting information: Dataset S1).

**Supplementary Information includes:**

**Figures S1 to S7**

***Supplementary Figures:***


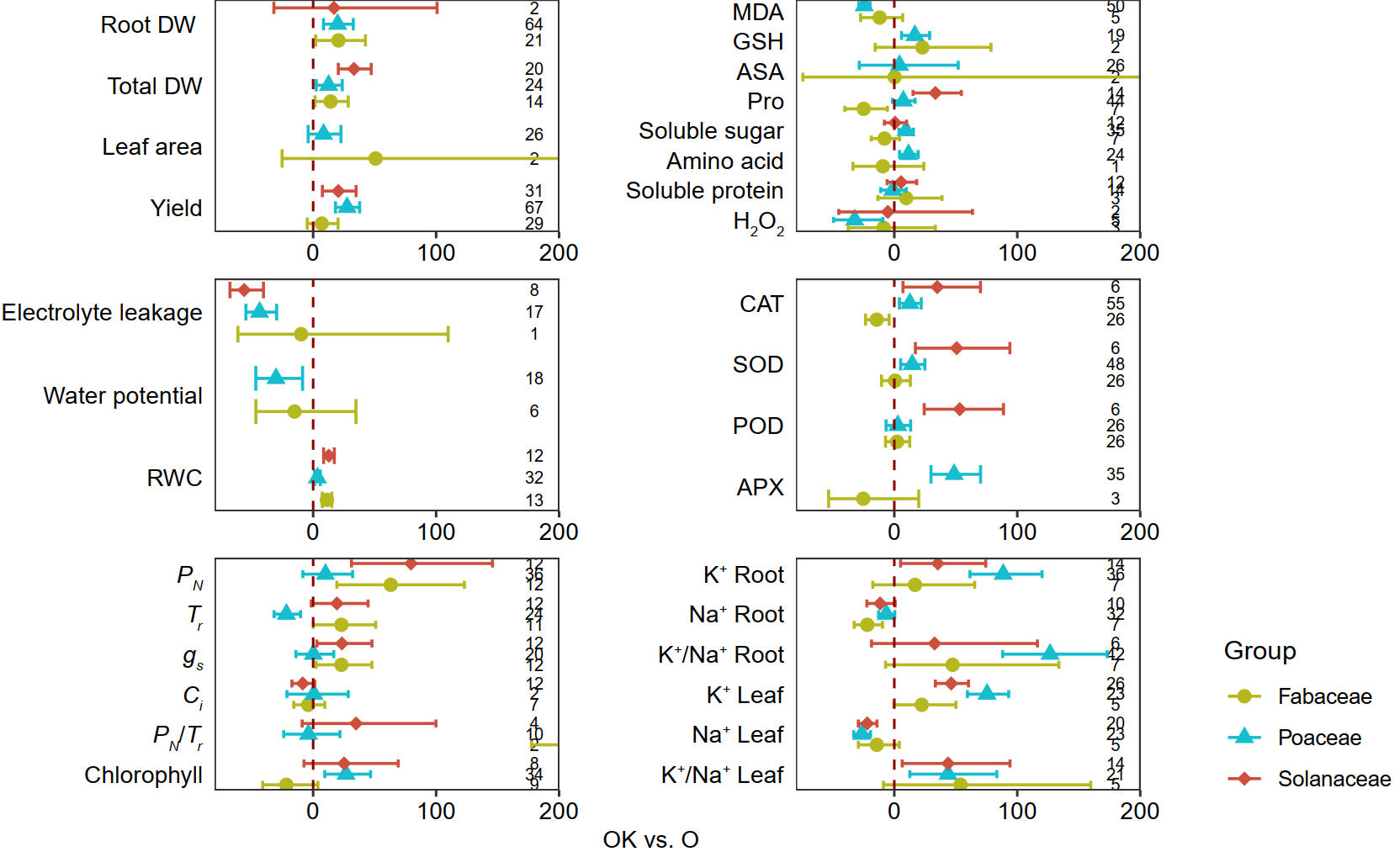


**Fig. S1** Comparisons of K effects on parameters involved in this meta‐analysis in Fabaceae, Poaceae and Solanaceae. under control and treatment conditions (OK vs. O). Values are means ± 95% confidence intervals. The number of observations is shown on the right side.

Abbreviations: RWC, leaf relative water content; GR, glutathione reductase; CAT, catalase; SOD, superoxide dismutase; POD, peroxidase; GPX, glutathione peroxidase; APX, ascorbate peroxidase; *P_N_*, photosynthetic rate; *T_r_*, transpiration rate; *g_s_*, stomatal conductivity; *C_i_*, intercellular CO_2_ content; *P_N_*/ *T_r_*, photosynthetic rate/ transpiration rate; MDA, malonaldehyde; GSH, glutathione; GSSG, oxidized glutathione; ASA, ascorbic acid; Pro, proline; H_2_O_2_, hydrogen peroxide; Leaf DW, leaf dry weight; Root DW, root dry weight; Total DW, total dry weight; K^+^ Root, root potassium content; Na^+^ Root, root sodium content; K^+^/Na^+^ Root, the ratio of potassium content to sodium content in root; K^+^ Leaf, leaf potassium content; Na^+^ Leaf, leaf sodium content; K^+^/Na^+^ Leaf, the ratio of potassium content to sodium content in leaf. The numerical values on the right side indicate the total count of examined cases.


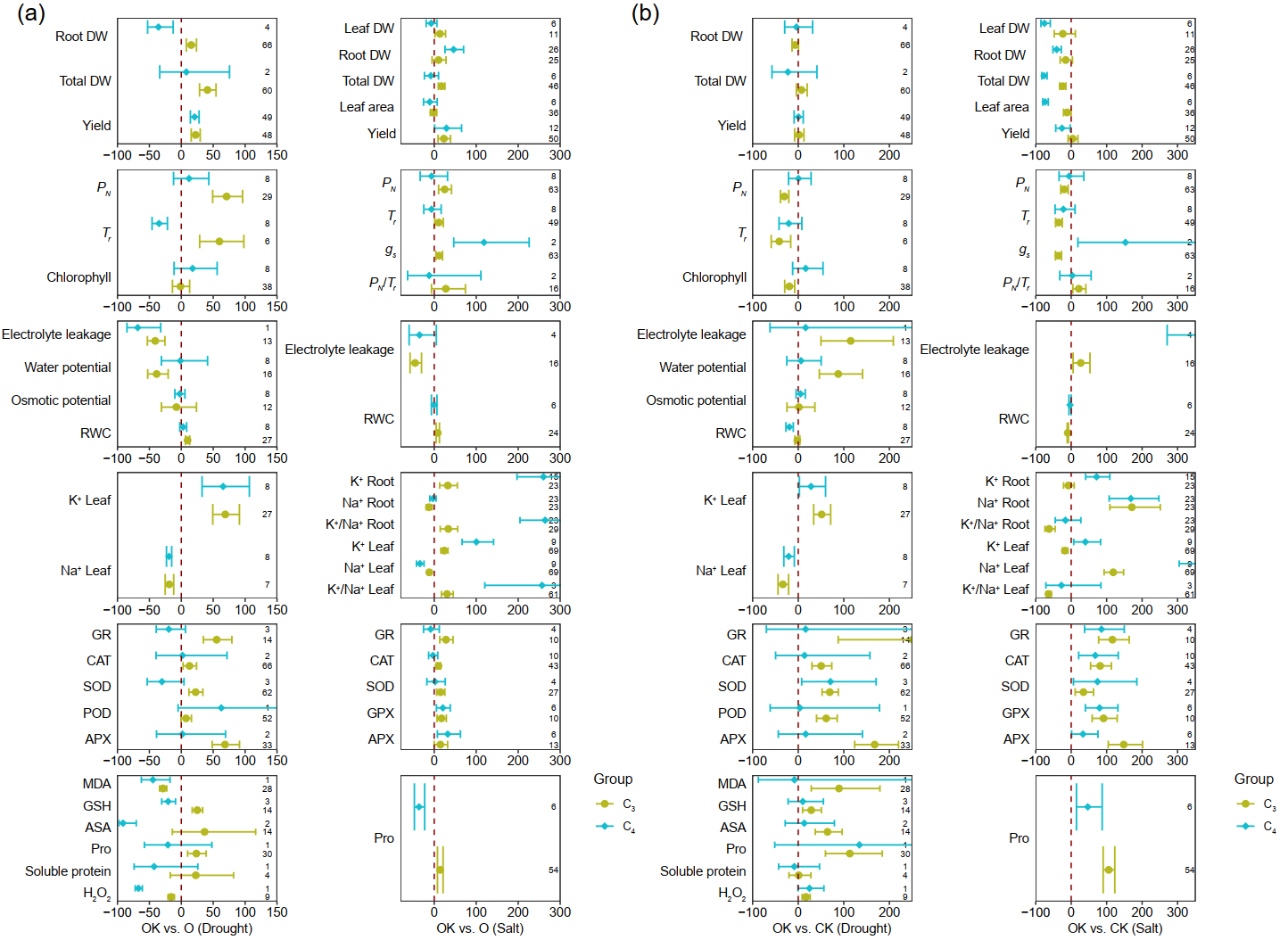


**Fig. S2** Responses of different photosynthetic types to potassium under osmotic stress. Comparisons of potassium effects on parameters involved in this meta‐analysis between C_3_ and C_4_ plants under control and treatment conditions (OK _(drought or salt)_ vs. O) (a). Comparisons of potassium effects on parameters involved in this meta‐analysis between C_3_ and C_4_ plants under control and treatment conditions (OK _(drought or salt)_ vs. CK) (b). Values are means ± 95% confidence intervals. The number of observations is shown on the right side.

Abbreviations: RWC, leaf relative water content; GR, glutathione reductase; CAT, catalase; SOD, superoxide dismutase; POD, peroxidase; GPX, glutathione peroxidase; APX, ascorbate peroxidase; *P_N_*, photosynthetic rate; *T_r_*, transpiration rate; *g_s_*, stomatal conductivity; *C_i_*, intercellular CO_2_ content; *P_N_*/ *T_r_*, photosynthetic rate/ transpiration rate; MDA, malonaldehyde; GSH, glutathione; GSSG, oxidized glutathione; ASA, ascorbic acid; Pro, proline; H_2_O_2_, hydrogen peroxide; Leaf DW, leaf dry weight; Root DW, root dry weight; Total DW, total dry weight; K^+^ Root, root potassium content; Na^+^ Root, root sodium content; K^+^/Na^+^ Root, the ratio of potassium content to sodium content in root; K^+^ Leaf, leaf potassium content; Na^+^ Leaf, leaf sodium content; K^+^/Na^+^ Leaf, the ratio of potassium content to sodium content in leaf. The numerical values on the right side indicate the total count of examined cases.


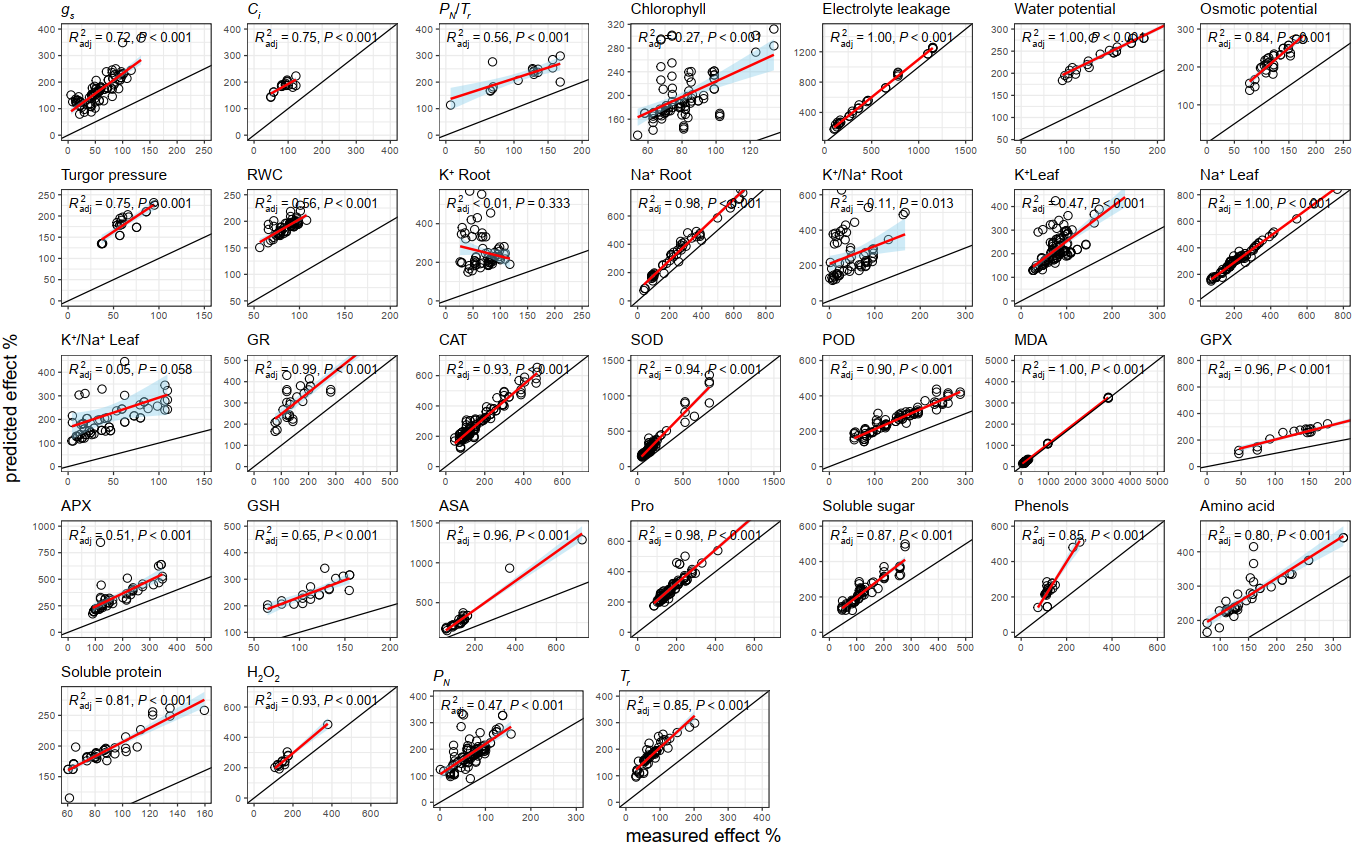


**Fig. S3** Relationship between observed (measured effect) and calculated (predicted effect) effects of combined exposure to K and osmotic stress for variables describing plant acclimation responses. The black line indicates a linear fit with a 1:1 correlation.

Abbreviations: RWC, leaf relative water content; GR, glutathione reductase; CAT, catalase; SOD, superoxide dismutase; POD, peroxidase; GPX, glutathione peroxidase; APX, ascorbate peroxidase; *P_N_*, photosynthetic rate; *T_r_*, transpiration rate; *g_s_*, stomatal conductivity; *C_i_*, intercellular CO_2_ content; *P_N_*/ *T_r_*, photosynthetic rate/ transpiration rate; MDA, malonaldehyde; GSH, glutathione; GSSG, oxidized glutathione; ASA, ascorbic acid; Pro, proline; H_2_O_2_, hydrogen peroxide; K^+^ Root, root potassium content; Na^+^ Root, root sodium content; K^+^/Na^+^ Root, the ratio of potassium content to sodium content in root; K^+^ Leaf, leaf potassium content; Na^+^ Leaf, leaf sodium content; K^+^/Na^+^ Leaf, the ratio of potassium content to sodium content in leaf.


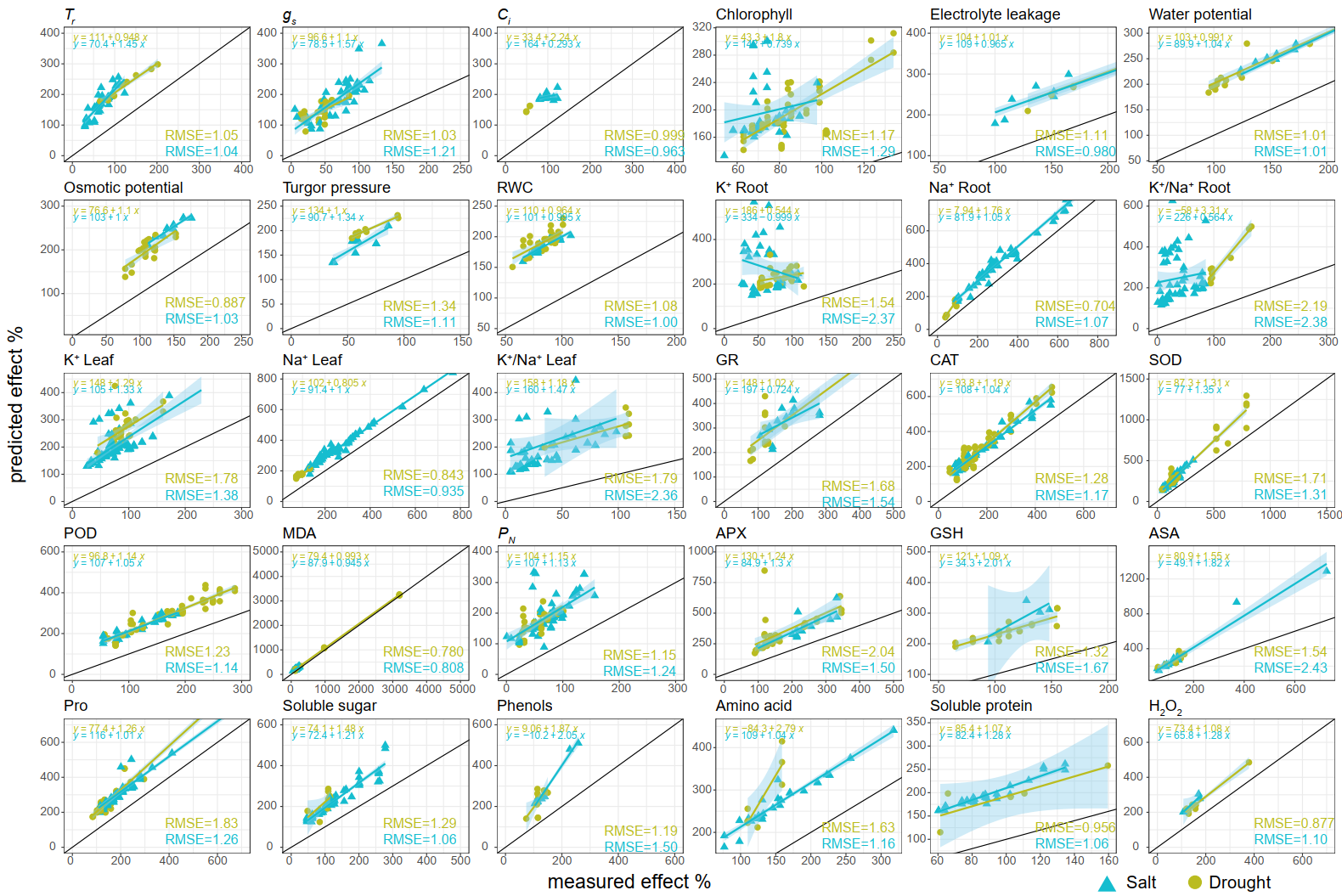


**Fig. S4** Relationship between observed (measured effect) and calculated (predicted effect) effects of combined exposure to K and osmotic stress for variables describing plant acclimation responses under different stress types. The black line indicates a linear fit with a 1:1 correlation. The 'RMSE' value in the fitted equation signifies the strength of the interaction.

Abbreviations: RWC, leaf relative water content; GR, glutathione reductase; CAT, catalase; SOD, superoxide dismutase; POD, peroxidase; APX, ascorbate peroxidase; *P_N_*, photosynthetic rate; *T_r_,* transpiration rate; *g_s_*, stomatal conductivity; *C_i_*, intercellular CO_2_ content; *P_N_*/ *T_r_*, photosynthetic rate/ transpiration rate; MDA, malonaldehyde; GSH, glutathione; GSSG, oxidized glutathione; ASA, ascorbic acid; Pro, proline; H_2_O_2_, hydrogen peroxide; K^+^ Root, root potassium content; Na^+^ Root, root sodium content; K^+^/Na^+^ Root, the ratio of potassium content to sodium content in root; K^+^ Leaf, leaf potassium content; Na^+^ Leaf, leaf sodium content; K^+^/Na^+^ Leaf, the ratio of potassium content to sodium content in leaf.


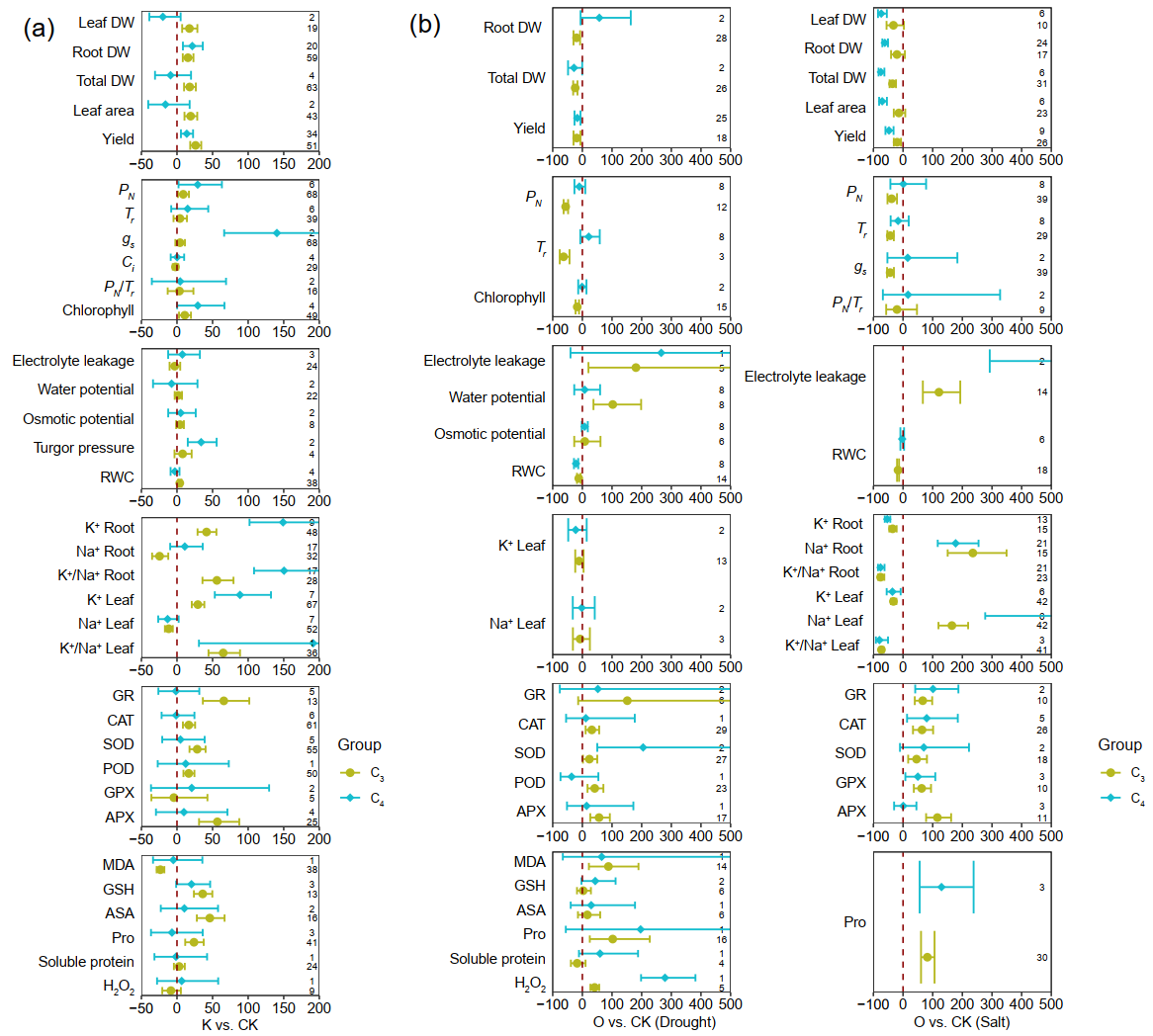


**Fig. S5** Comparisons of K effects on parameters involved in plant meta‐analysis under control and conditions (K vs. CK) (a). Comparisons of K effects on parameters involved in this meta‐analysis between C_3_ and C_4_ plants under control and treatment conditions (O _(drought or salt)_ vs. CK) (b). Values are means ± 95% confidence intervals. The number of observations is shown on the right side.

Abbreviations: RWC, leaf relative water content; GR, glutathione reductase; CAT, catalase; SOD, superoxide dismutase; POD, peroxidase; GPX, glutathione peroxidase; APX, ascorbate peroxidase; *P_N_*, photosynthetic rate; *T_r_*, transpiration rate; *g_s_*, stomatal conductivity; *C_i_*, intercellular CO_2_ content; *P_N_*/ *T_r_*, photosynthetic rate/ transpiration rate; MDA, malonaldehyde; GSH, glutathione; GSSG, oxidized glutathione; ASA, ascorbic acid; Pro, proline; H_2_O_2_, hydrogen peroxide; Leaf DW, leaf dry weight; Root DW, root dry weight; Total DW, total dry weight; K^+^ Root, root potassium content; Na^+^ Root, root sodium content; K^+^/Na^+^ Root, the ratio of potassium content to sodium content in root; K^+^ Leaf, leaf potassium content; Na^+^ Leaf, leaf sodium content; K^+^/Na^+^ Leaf, the ratio of potassium content to sodium content in leaf. The numerical values on the right side indicate the total count of examined cases.


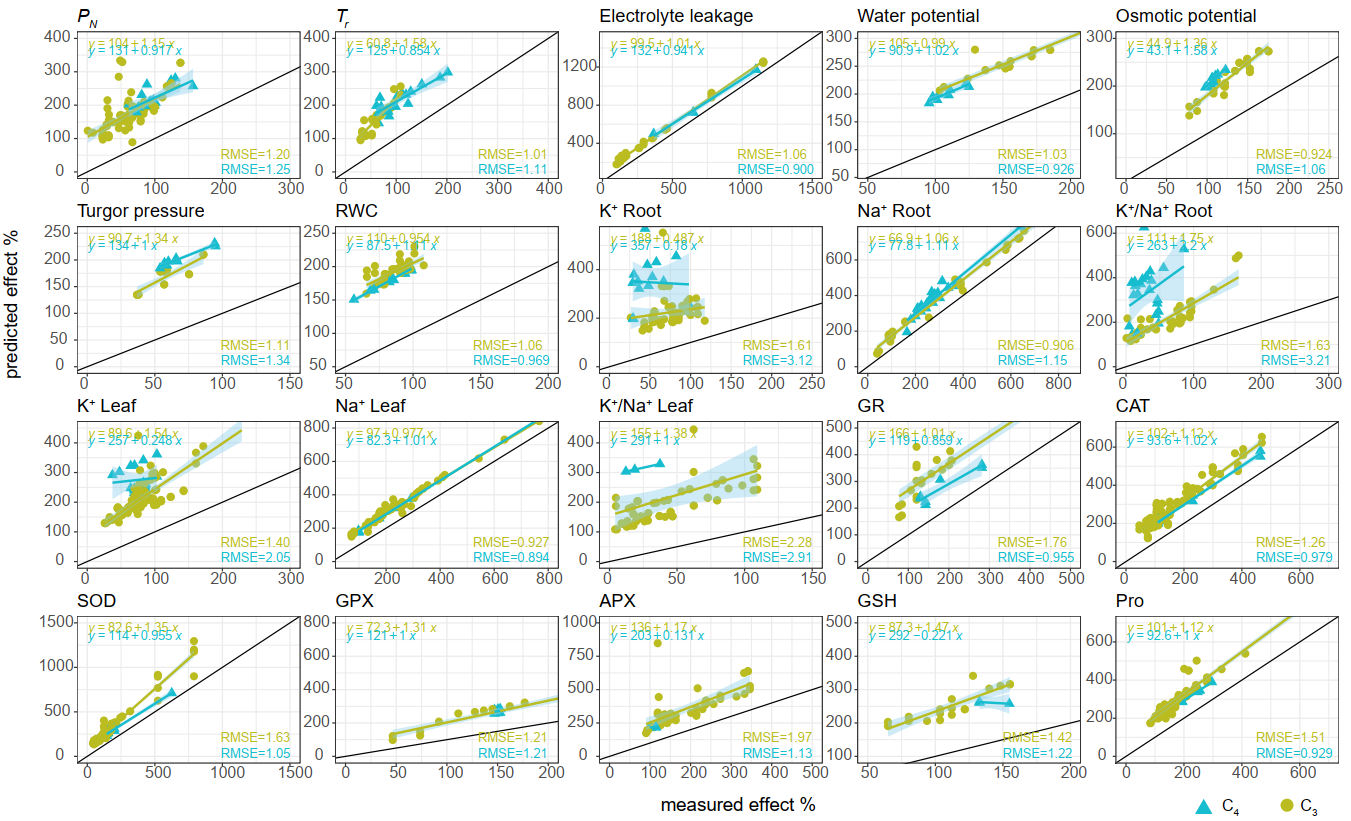


**Fig. S6** Relationship between observed (measured effect) and calculated (predicted effect) effects of combined exposure to K and osmotic stress for variables describing plant acclimation responses under different plant photosynthetic types. The black line indicates a linear fit with a 1:1 correlation. The 'RMSE' value in the fitted equation signifies the strength of the interaction.

Abbreviations: RWC, leaf relative water content; GR, glutathione reductase; CAT, catalase; SOD, superoxide dismutase; POD, peroxidase; GPX, glutathione peroxidase; APX, ascorbate peroxidase; *P_N_*, photosynthetic rate; *T_r_*, transpiration rate; *g_s_*, stomatal conductivity; *C_i_*, intercellular CO_2_ content; *P_N_*/ *T_r_*, photosynthetic rate/ transpiration rate; MDA, malonaldehyde; GSH, glutathione; GSSG, oxidized glutathione; ASA, ascorbic acid; Pro, proline; H_2_O_2_, hydrogen peroxide; K^+^ Root, root potassium content; Na^+^ Root, root sodium content; K^+^/Na^+^ Root, the ratio of potassium content to sodium content in root; K^+^ Leaf, leaf potassium content; Na^+^ Leaf, leaf sodium content; K^+^/Na^+^ Leaf, the ratio of potassium content to sodium content in leaf.


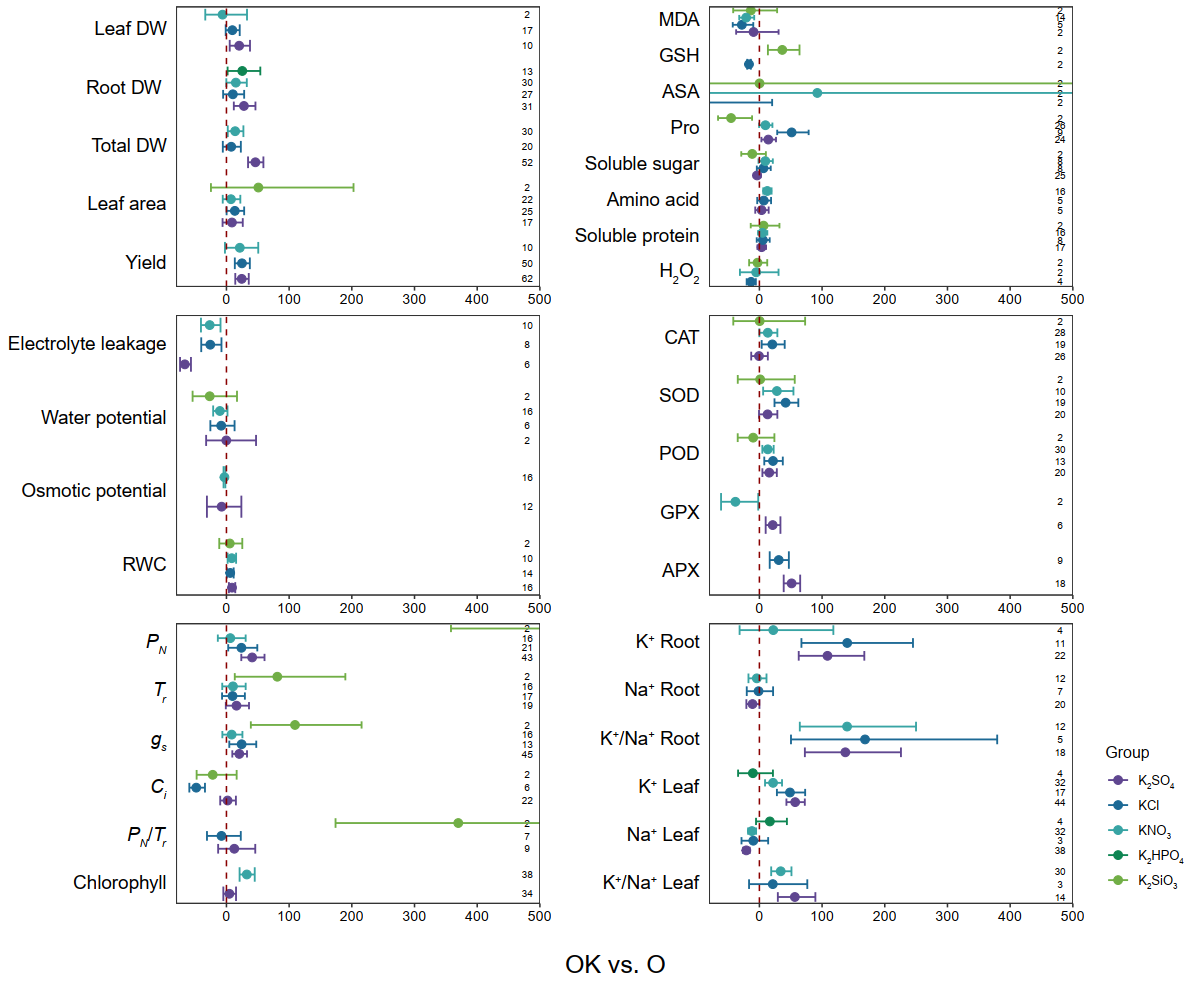


**Fig. S7** Comparisons of K effects on parameters involved in this meta‐analysis in K_2_HPO_4_, K_2_SO_4_, KCl, KNO_3_ and K_2_SiO_3_ under control and treatment conditions (OK vs. O). Values are means ± 95% confidence intervals. The number of observations is shown on the right side.

Abbreviations: RWC, leaf relative water content; GR, glutathione reductase; CAT, catalase; SOD, superoxide dismutase; POD, peroxidase; GPX, glutathione peroxidase; APX, ascorbate peroxidase; *P_N_*, photosynthetic rate; *T_r_*, transpiration rate; *g_s_*, stomatal conductivity; *C_i_*, intercellular CO_2_ content; *P_N_*/ *T_r_*, photosynthetic rate/ transpiration rate; MDA, malonaldehyde; GSH, glutathione; GSSG, oxidized glutathione; ASA, ascorbic acid; Pro, proline; H_2_O_2_, hydrogen peroxide; Leaf DW, leaf dry weight; Root DW, root dry weight; Total DW, total dry weight; K^+^ Root, root potassium content; Na^+^ Root, root sodium content; K^+^/Na^+^ Root, the ratio of potassium content to sodium content in root; K^+^ Leaf, leaf potassium content; Na^+^ Leaf, leaf sodium content; K^+^/Na^+^ Leaf, the ratio of potassium content to sodium content in leaf. The numerical values on the right side indicate the total count of examined cases.
