# Supplementary figures and images for: Applied potassium negates osmotic stress impacts on plant physiological processes: a meta-analysis
Source: Hortic Res. 2024 Nov 18;12(2):uhae318. doi: 10.1093/hr/uhae318 (PMC11825146; doi:10.1093/hr/uhae318)

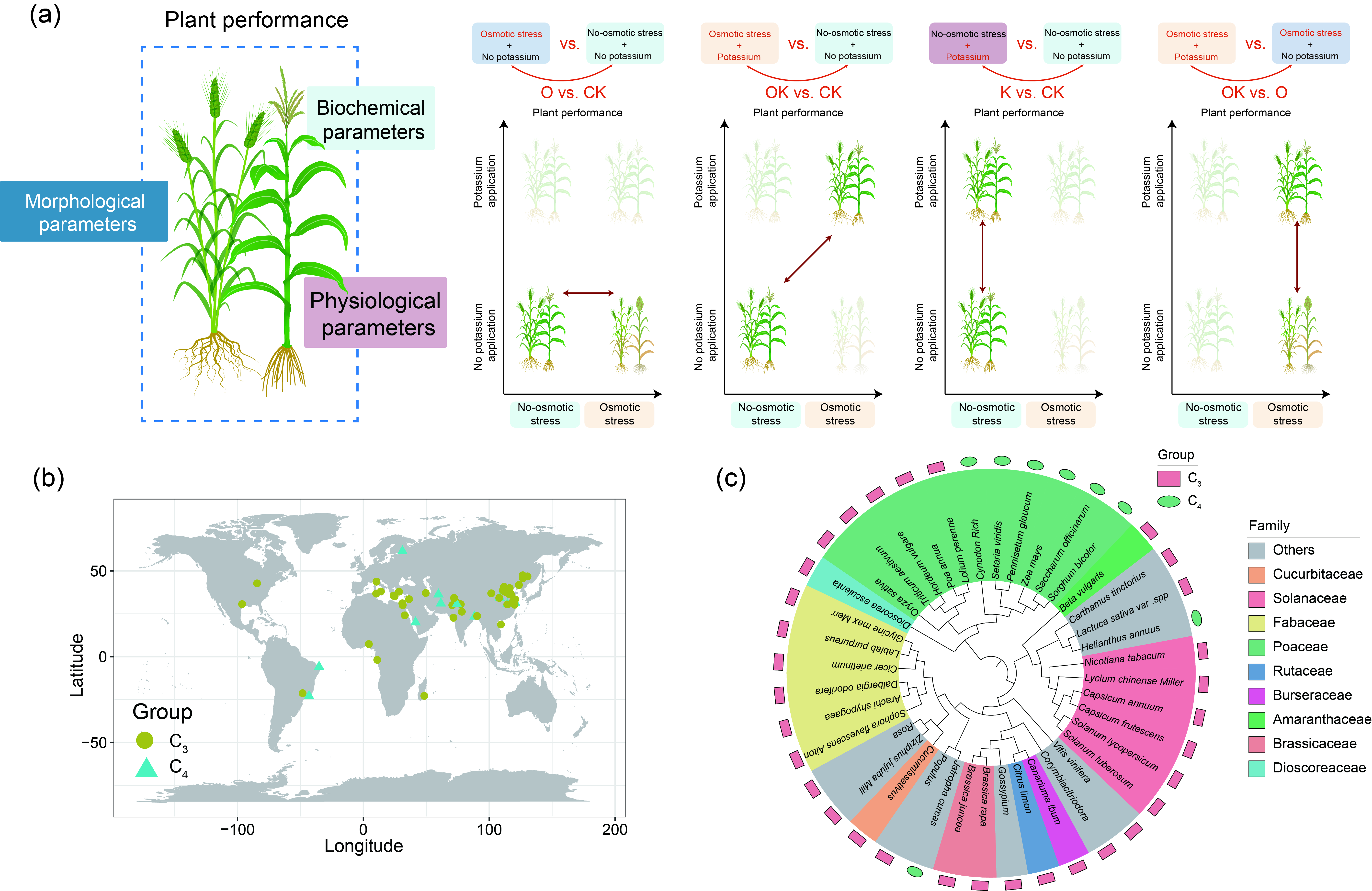

Supplement: Web_Material_uhae318 [file web_material_uhae318.zip › Fig. 1.tif]

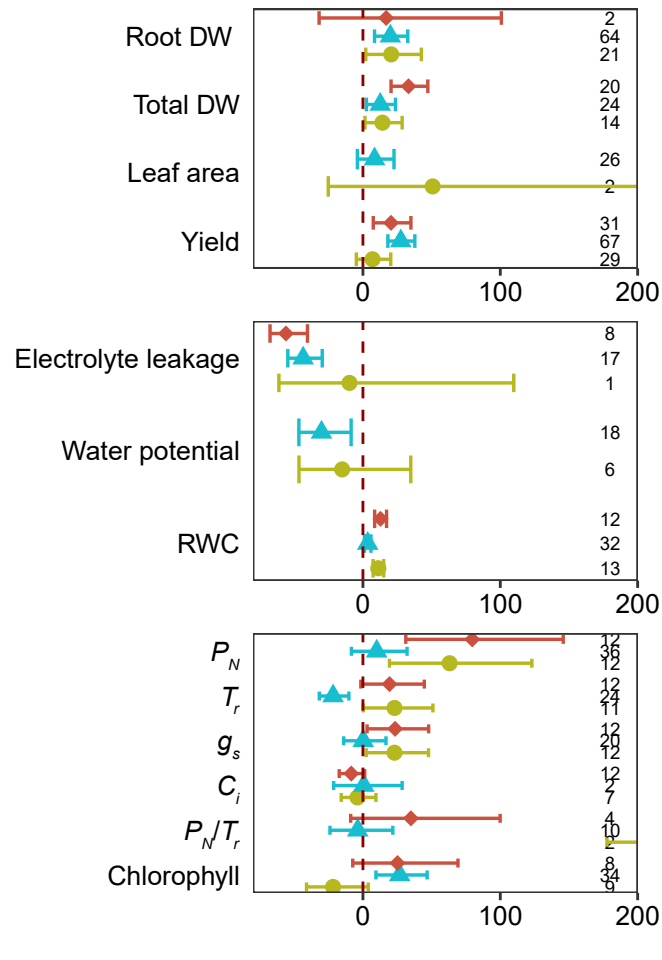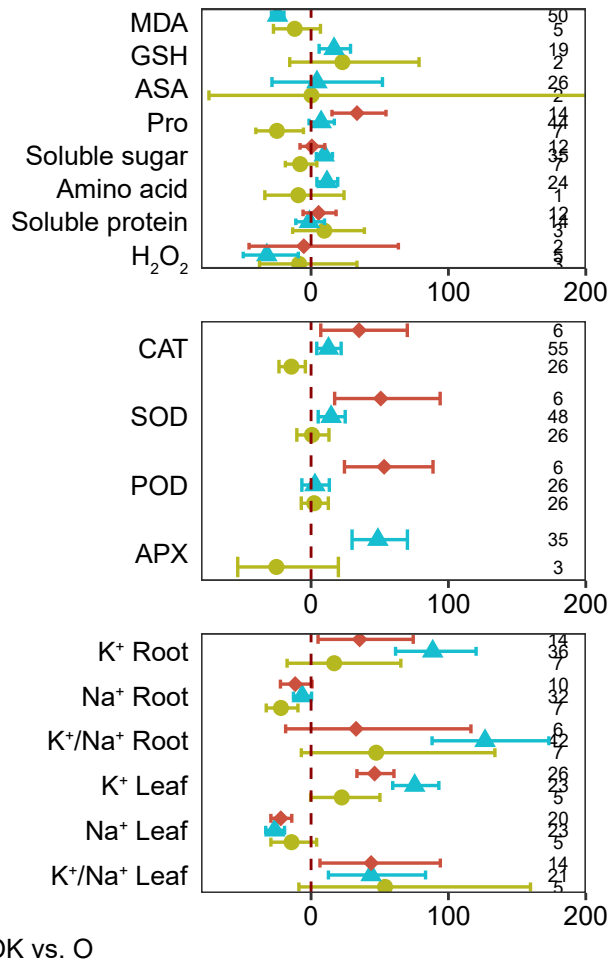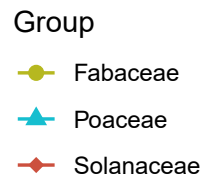

Supplement: Web_Material_uhae318 [file web_material_uhae318.zip › Fig. S1.pdf]

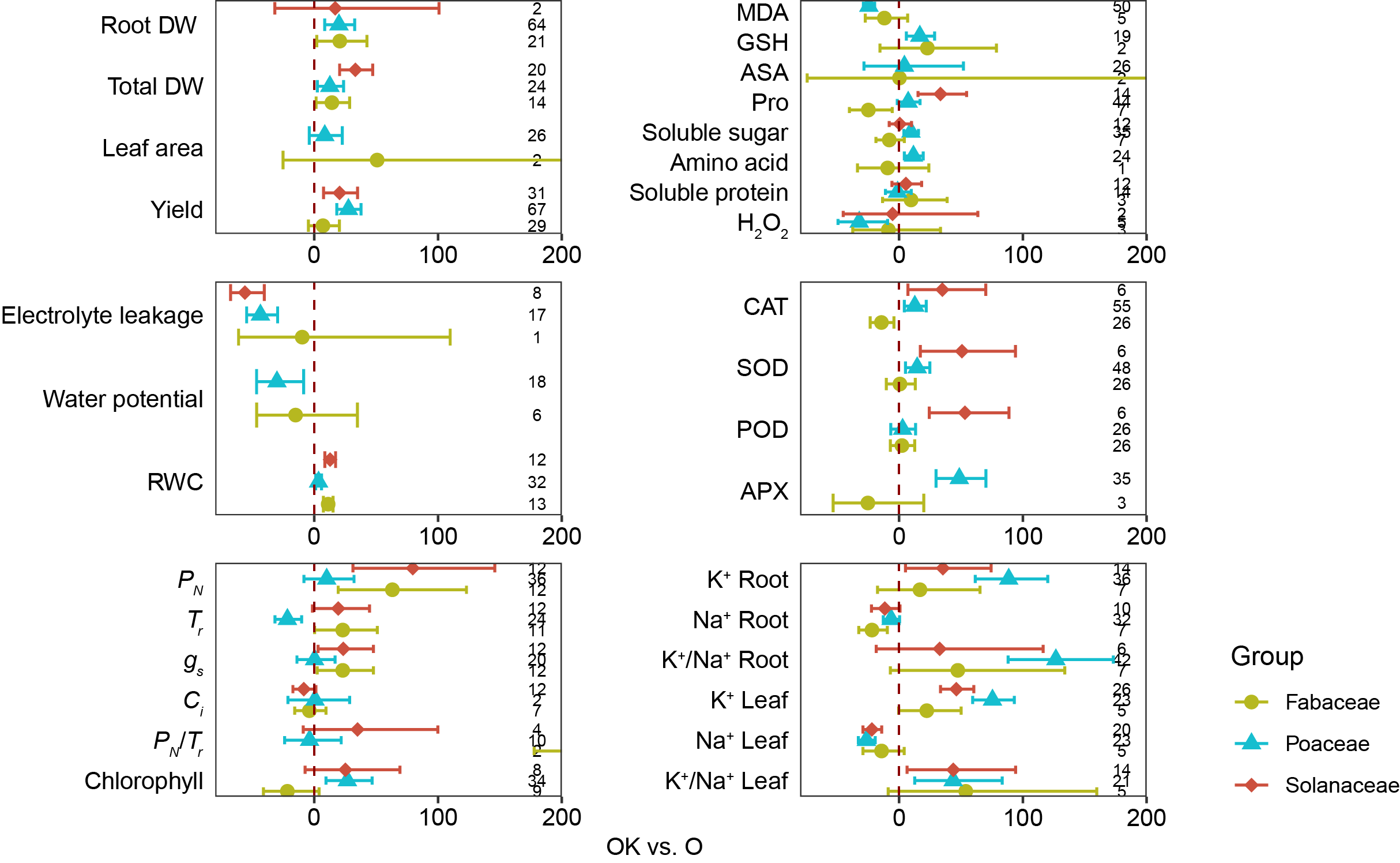

Supplement: Web_Material_uhae318 [file web_material_uhae318.zip › Fig. S1.tif]

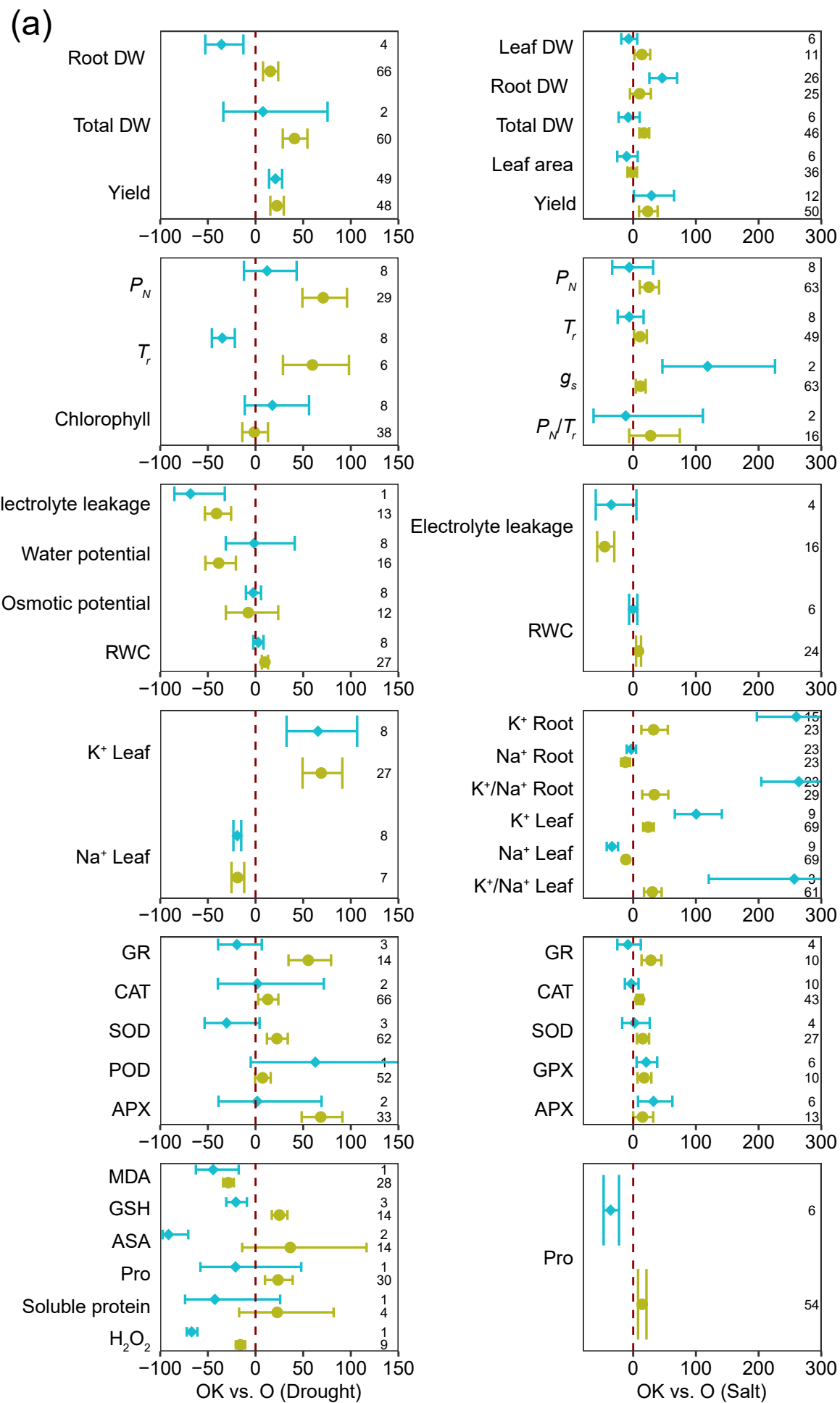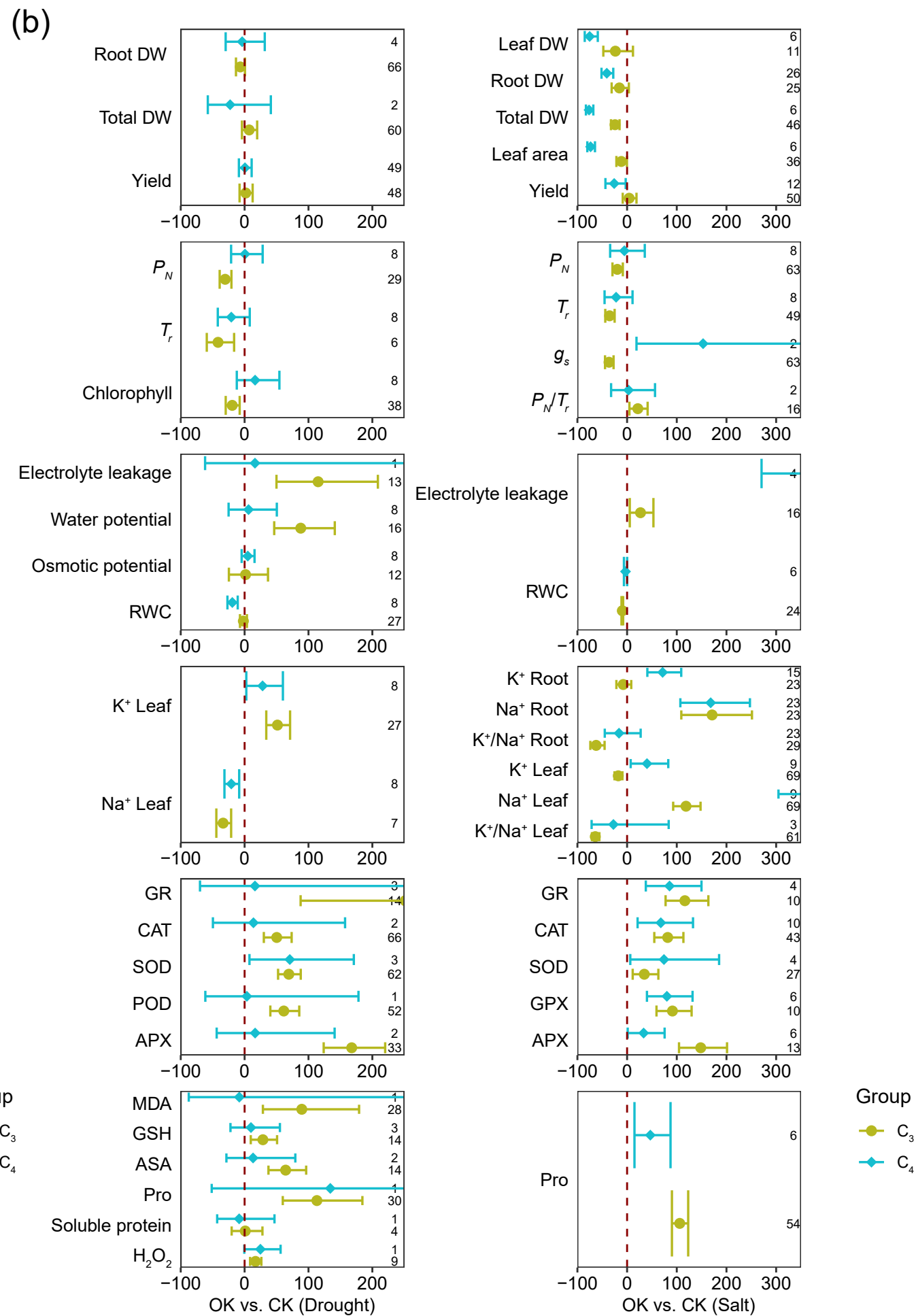

Supplement: Web_Material_uhae318 [file web_material_uhae318.zip › Fig. S2.pdf]

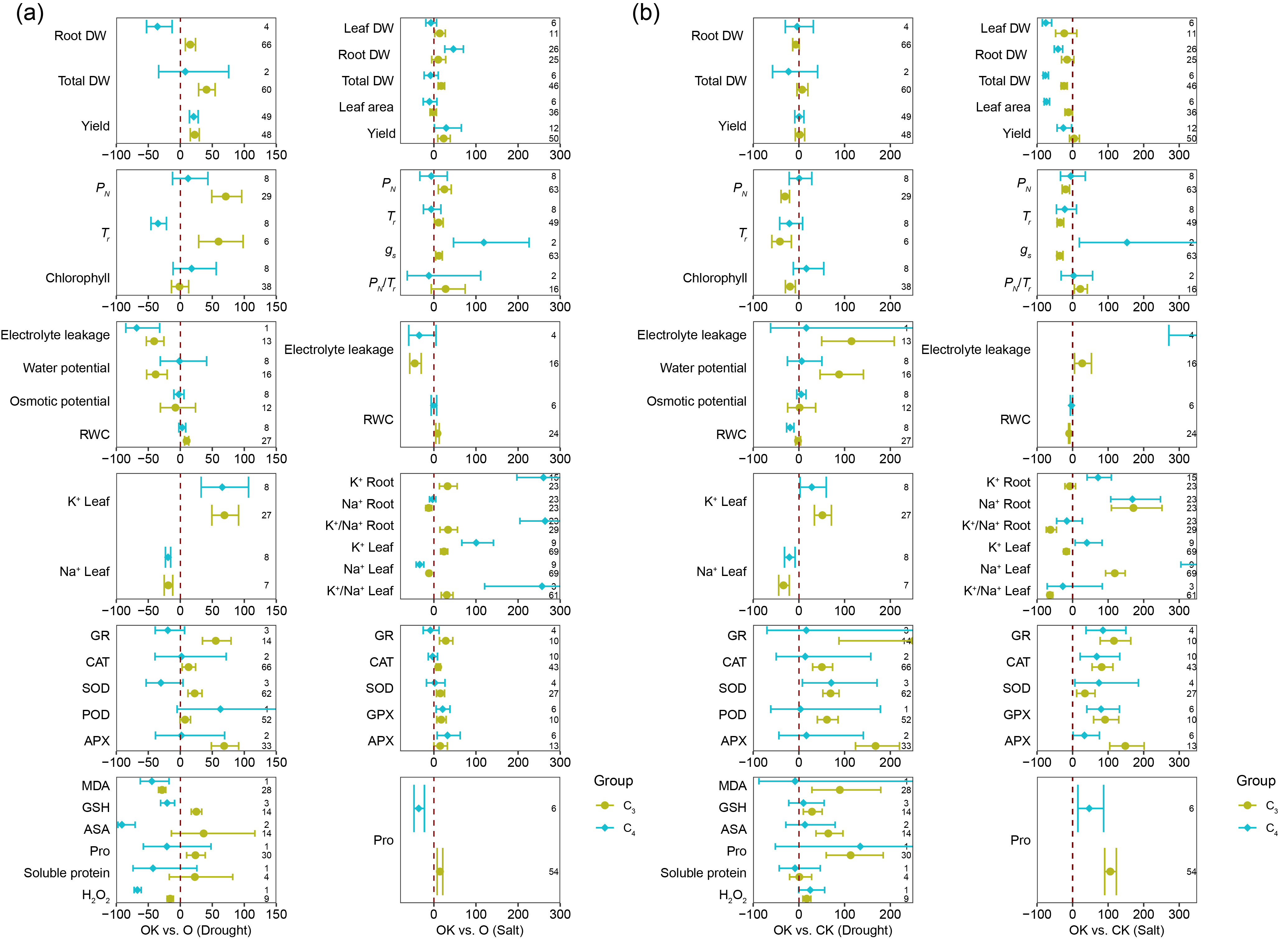

Supplement: Web_Material_uhae318 [file web_material_uhae318.zip › Fig. S2.tif]

predicted effect %

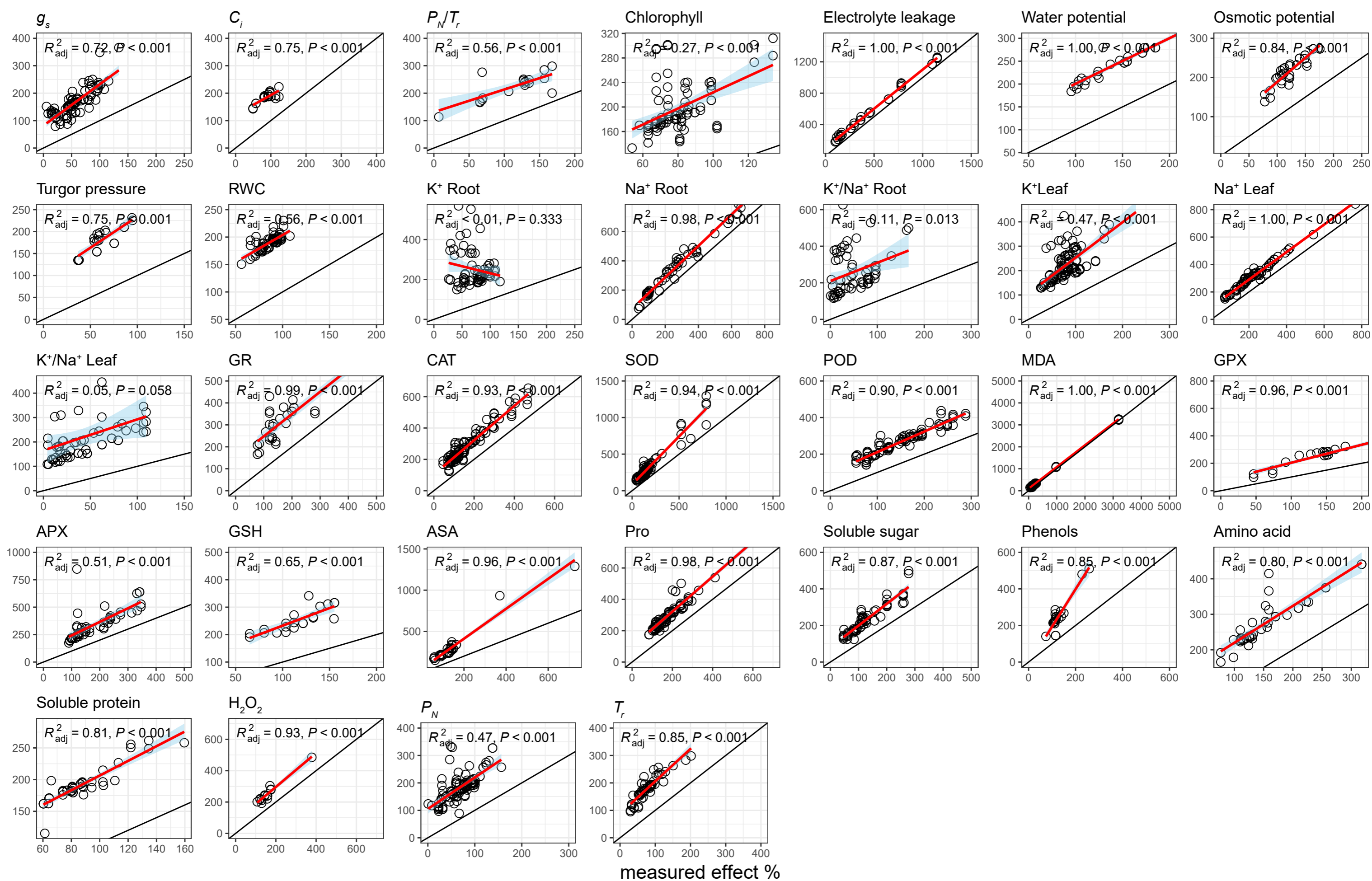

Supplement: Web_Material_uhae318 [file web_material_uhae318.zip › Fig. S3.pdf]

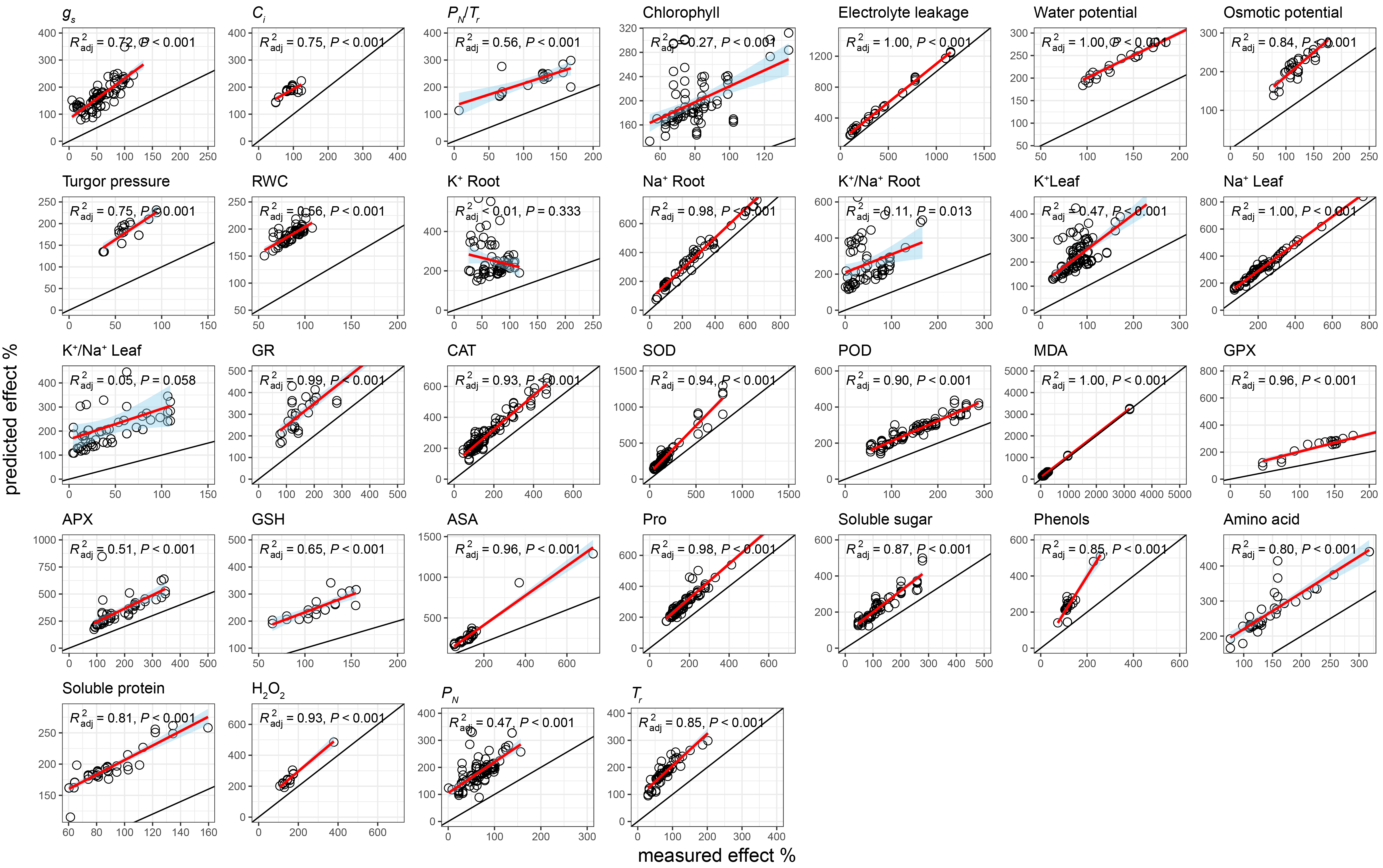

Supplement: Web_Material_uhae318 [file web_material_uhae318.zip › Fig. S3.tif]

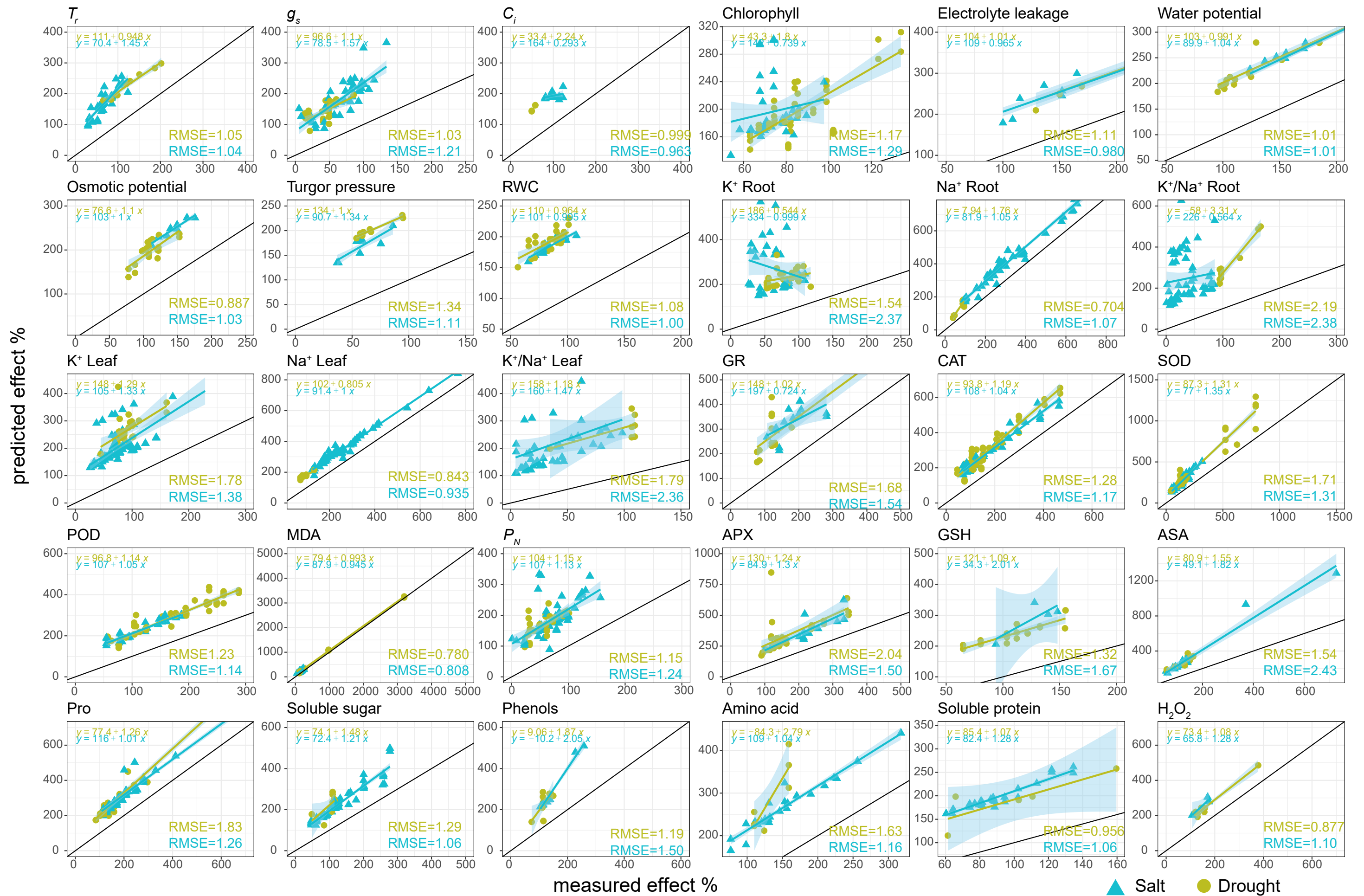

Supplement: Web_Material_uhae318 [file web_material_uhae318.zip › Fig. S4.pdf]

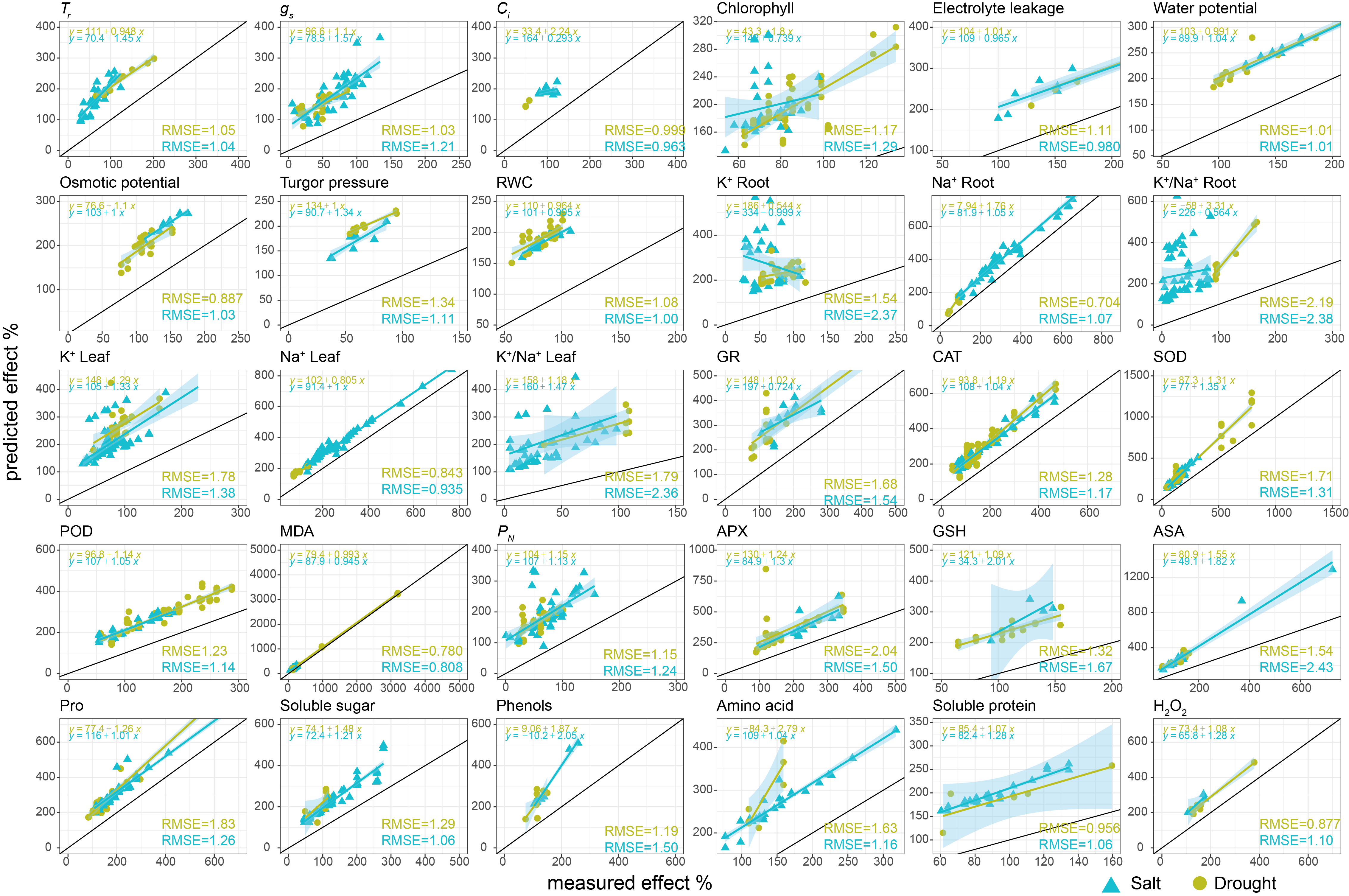

Supplement: Web_Material_uhae318 [file web_material_uhae318.zip › Fig. S4.tif]

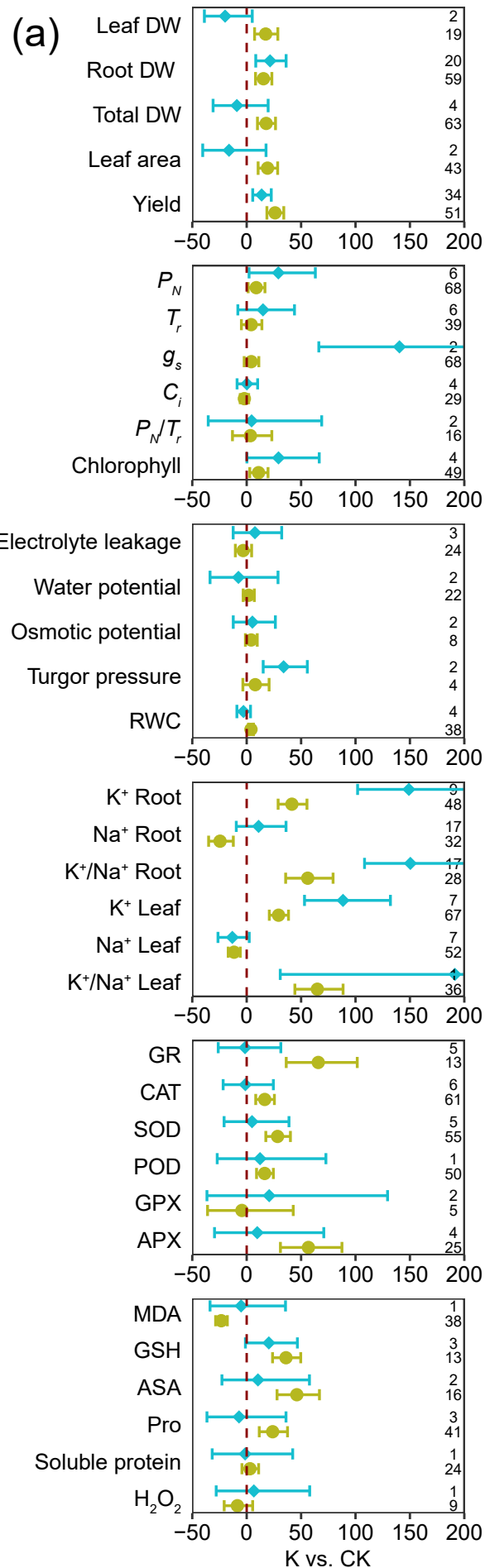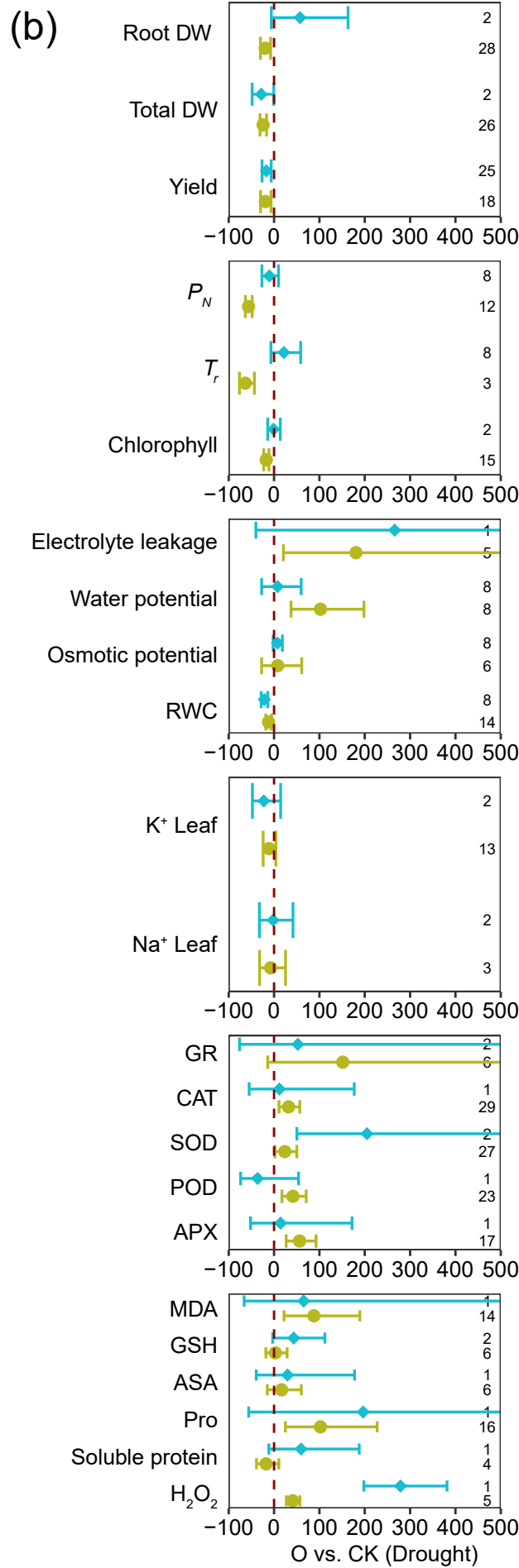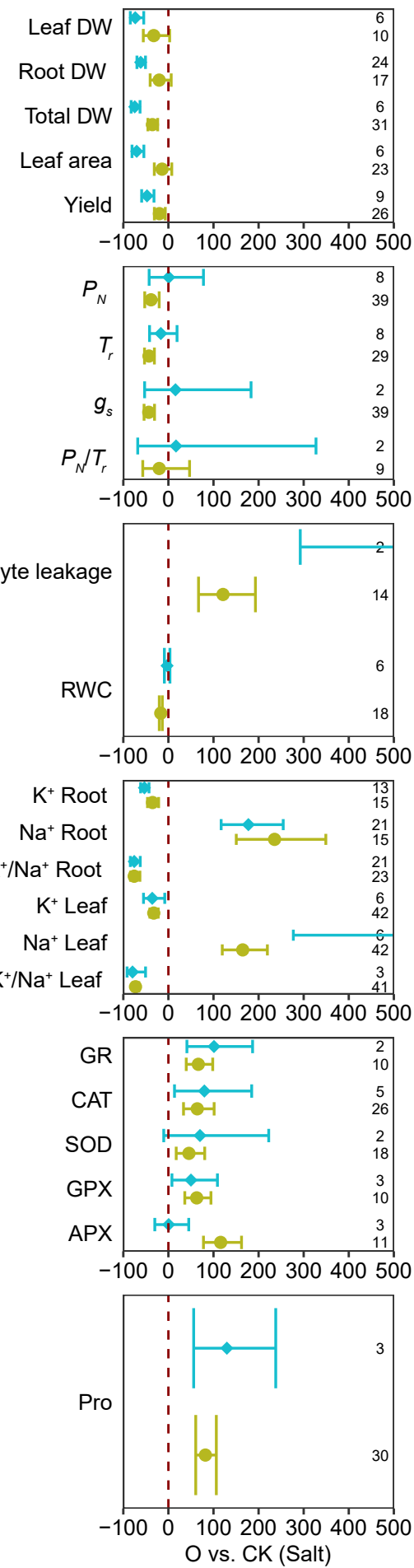

Supplement: Web_Material_uhae318 [file web_material_uhae318.zip › Fig. S5.pdf]

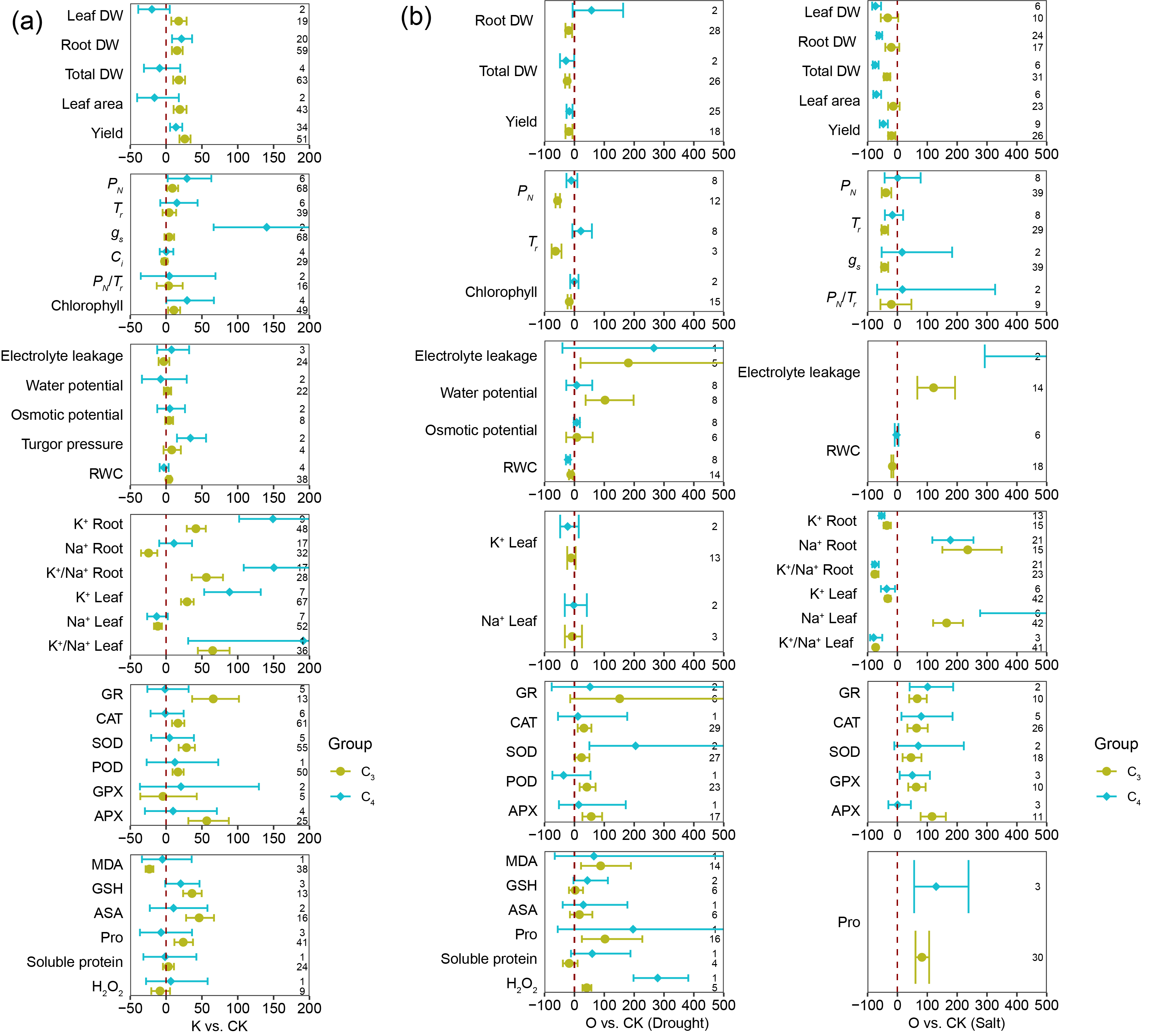

Supplement: Web_Material_uhae318 [file web_material_uhae318.zip › Fig. S5.tif]

predicted effect %

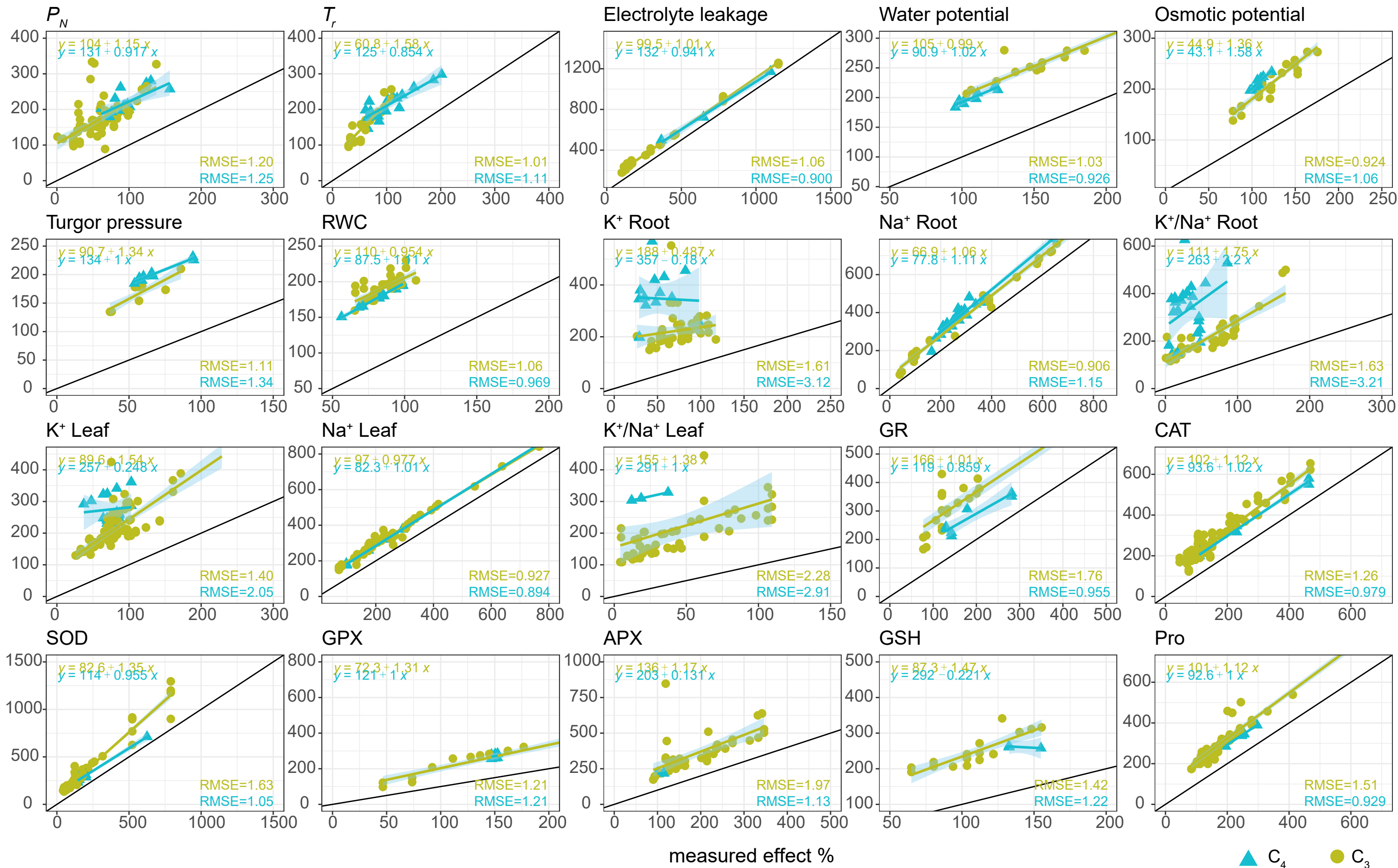

Supplement: Web_Material_uhae318 [file web_material_uhae318.zip › Fig. S6.pdf]

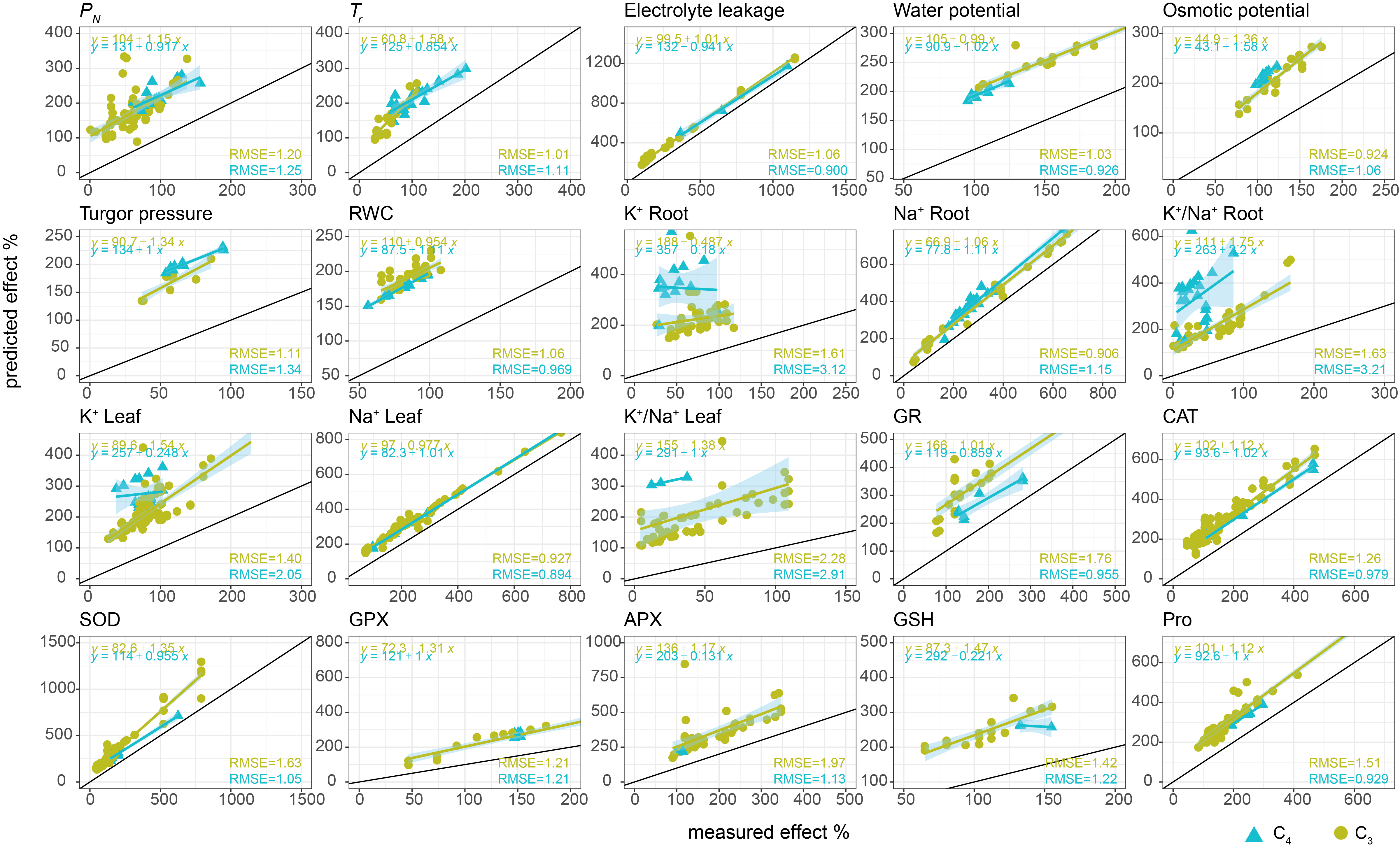

Supplement: Web_Material_uhae318 [file web_material_uhae318.zip › Fig. S6.tif]

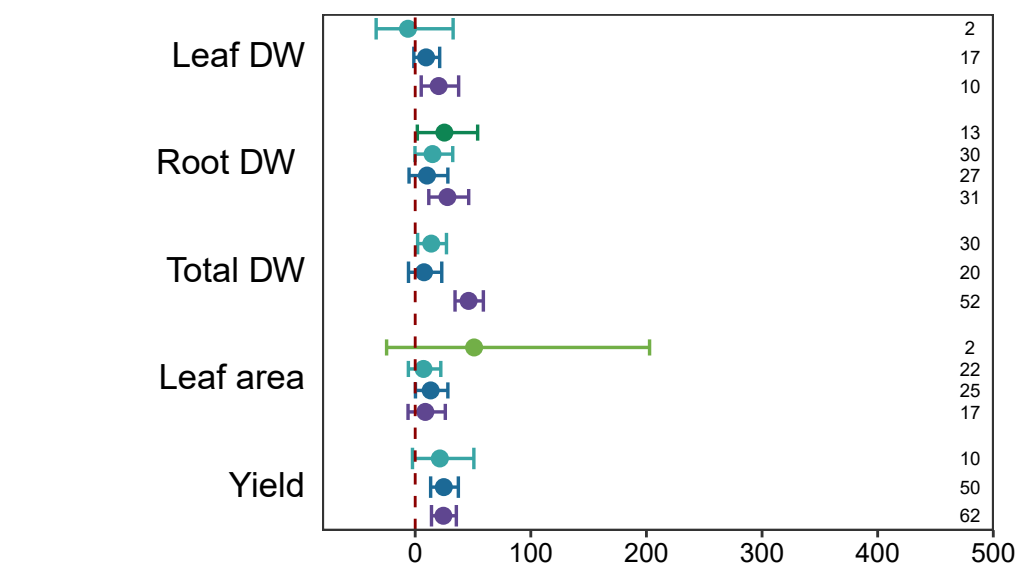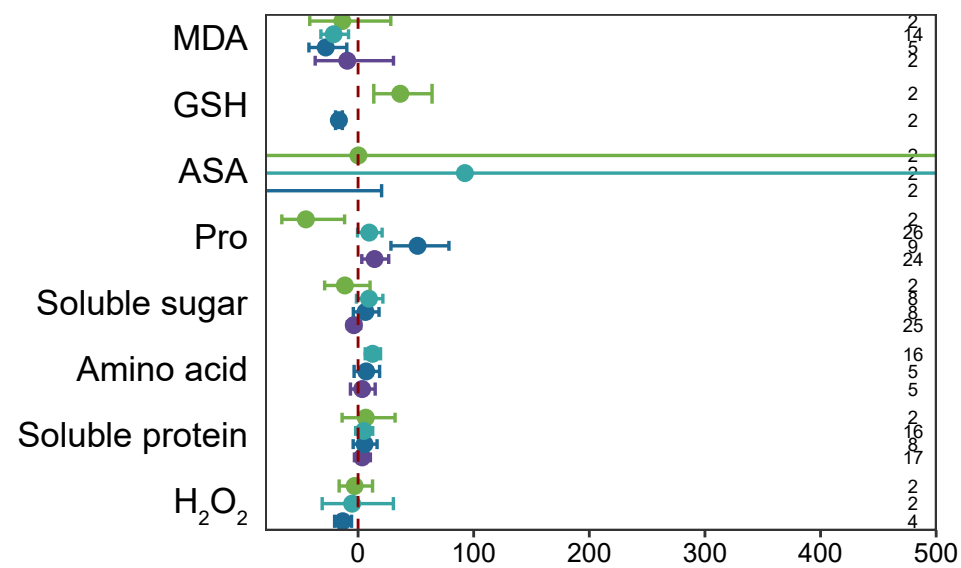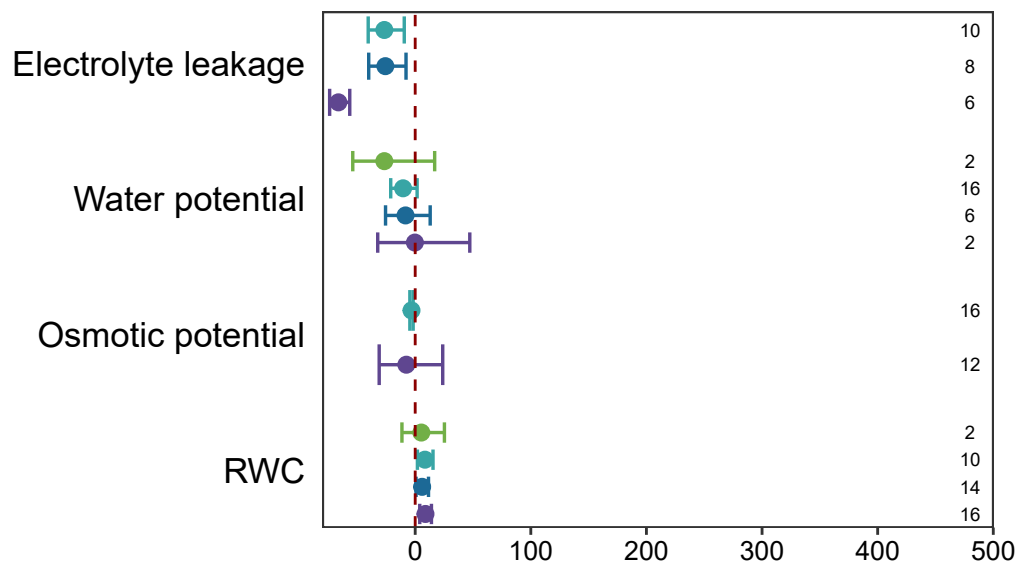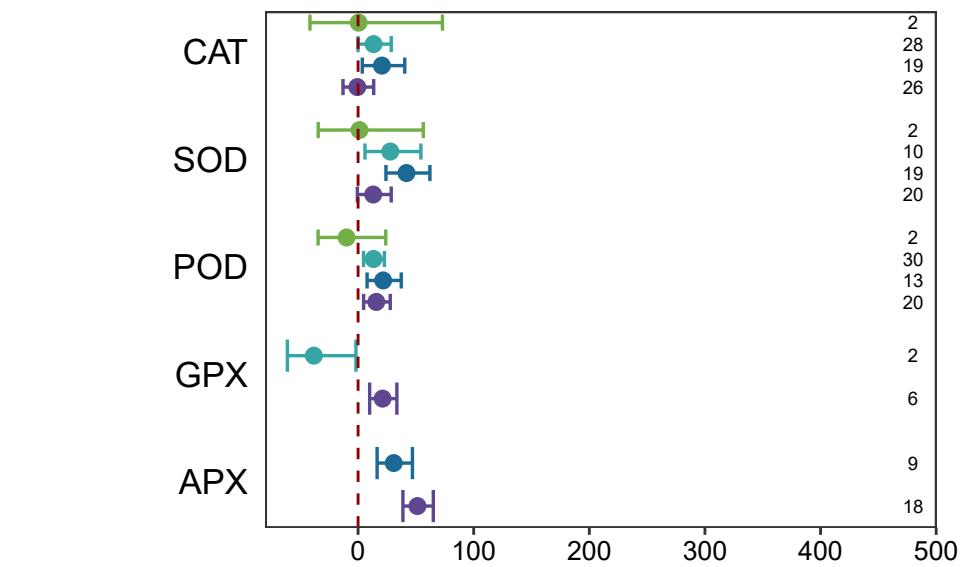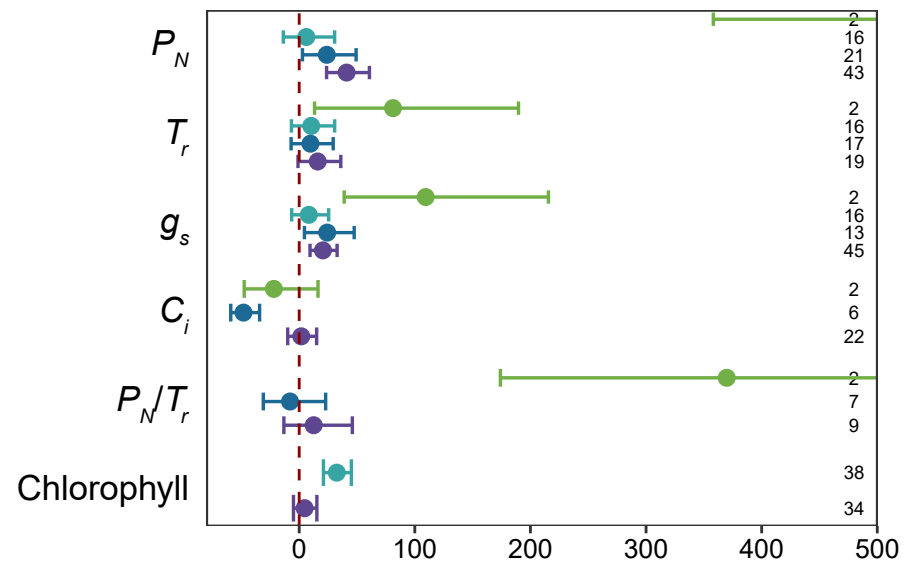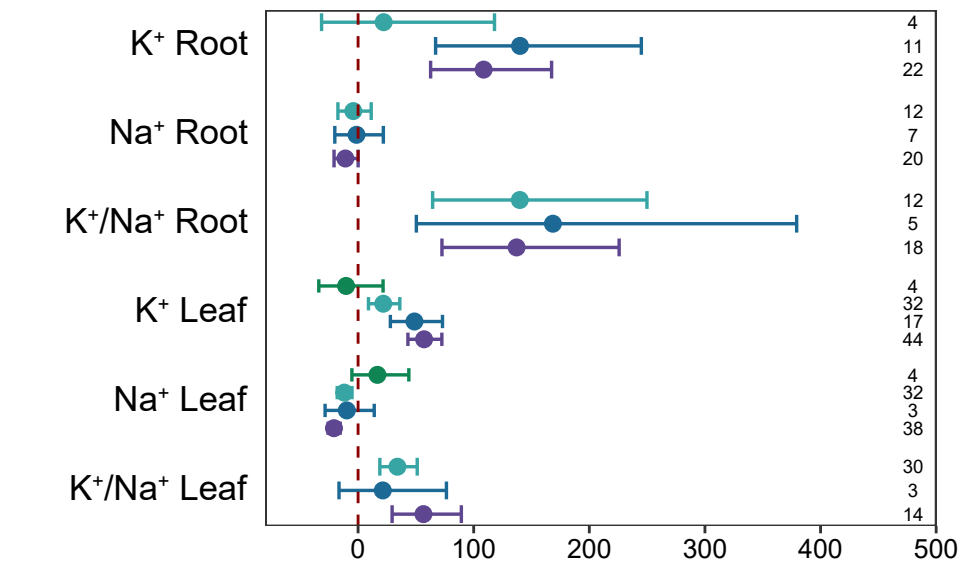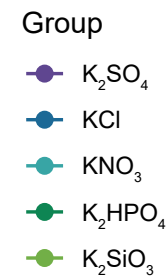

OK vs. O

Supplement: Web_Material_uhae318 [file web_material_uhae318.zip › Fig. S7.pdf]

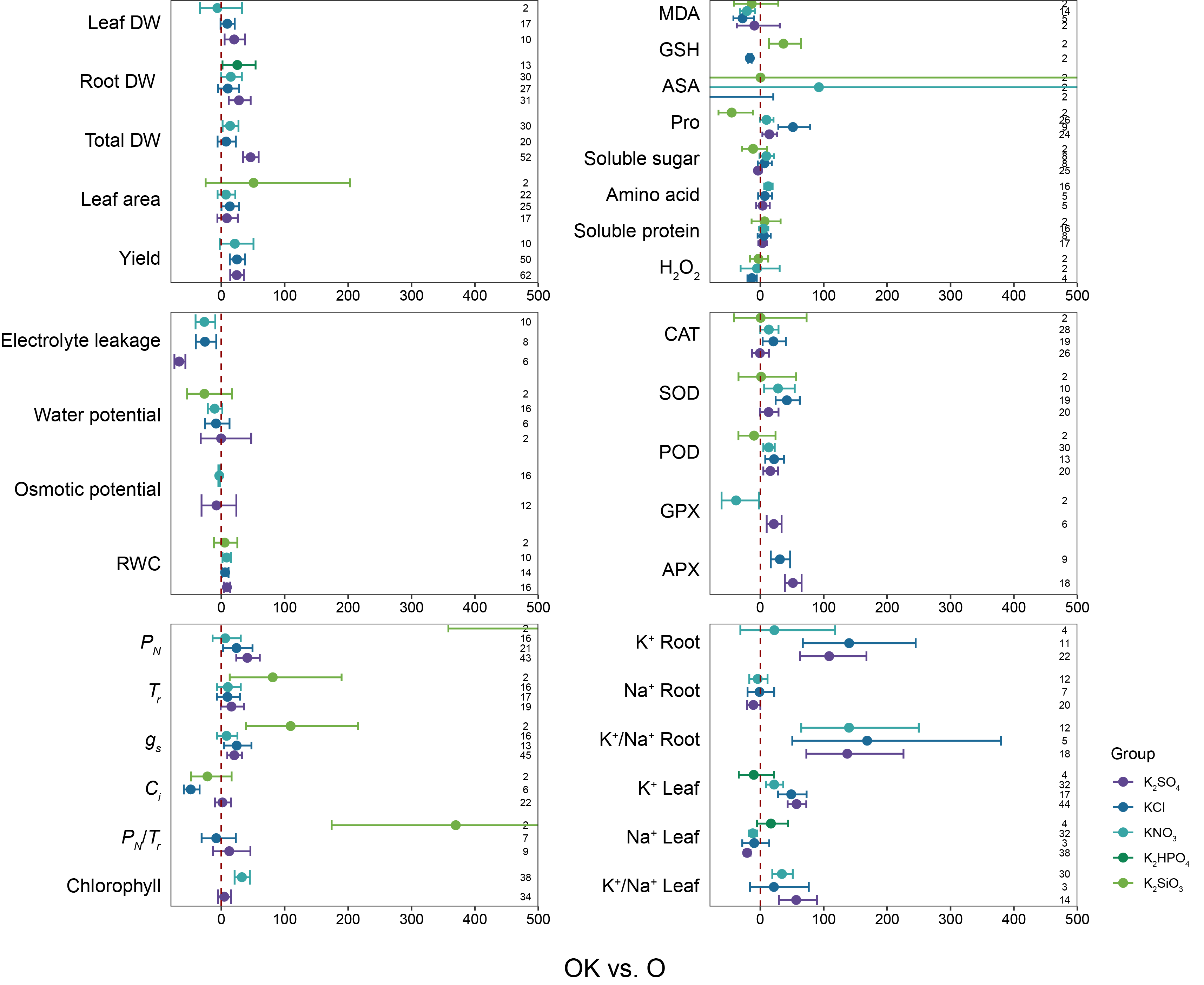

Supplement: Web_Material_uhae318 [file web_material_uhae318.zip › Fig. S7.tif]
